# Sensing Heat, Finding Cool: The Search for Water in Summertime Paris, New York, and London, 1880–1930

**DOI:** 10.1093/jsh/shaf109

**Published:** 2026-01-20

**Authors:** Jon Winder, Chloé Duteil, Kara Schlichting, Chris Pearson

## Abstract

At the turn of the twentieth century, Paris, New York, and London were epicenters of urban modernity, but these cities and their inhabitants were ill-equipped to cope with summer heat. Urbanites experienced thermal discomfort directly through their personal sensory registers, and the search for cooling water was a major, although overlooked, aspect of collective urban life in summer. Drawing on sensory and environmental history, and on the archives of city authorities and popular depictions of social life, we find that obtaining watery relief from the heat was limited by inequitable access to cooling infrastructures, including municipal and philanthropic baths and pools, which were often insufficient to meet urbanites’ needs. Instead, Parisians, New Yorkers, and Londoners turned to informal and often illicit methods to overcome the thermal inequalities that were baked into the brick and stone of their cities. Keeping cool in the melting metropolis was a challenge that demanded resourcefulness, bravery, and a willingness to disregard the rules and social norms that tried to regulate the use of other watery infrastructures, including ponds, fountains, hydrants, rivers, and canals. As present-day city authorities develop local adaptation plans in response to the global climate crisis, the fact that the coolness of water did not come easily or equally to turn-of-the-century urbanites should be at the forefront of our minds.

Paris, New York, and London have long been ill-equipped to cope with extreme summer weather. Residents suffered unequally when summer heated their bodies and homes in cities that were not built with high temperatures in mind. Neither Paris, New York, nor London featured summer-sensitive design in their architecture or layouts. The French newspaper *Le Monde Illustré* declared in summer 1884 that “Paris was not made for this season: there, it is possible to protect oneself from the cold, but it is impossible to escape the heat.”^1^ In New York, “the intensest [sic] hot weather” came between June and September, peaking in August. Alas, “[n]o one studie[d] how it should be met, but all suffer[ed] immeasurably.”^2^ This lack of attention to summer weather marked British cities, too. “London and London customs are ill-adapted to great heat” one newspaper reporter noted. The city’s inhabitants found themselves “languid and gasping.”^3^ Yet all thermal comfort was not lost; some individual and collective relief was possible. Water offered an essential way of finding coolness in the sweltering city.

The dramatic growth of Paris, New York, and London exacerbated summer discomfort. These three cities sweltered in summer even as they stood as epicenters of Western modernity.^4^ All had expanded rapidly in the nineteenth century, in ways that changed residents’ experiences of hot weather. Encroaching development turned accessible water bodies and waterfronts into industrial and commercial spaces rather than cooling respites. Landscapes once dominated by vegetation and tree canopy became sidewalks, streets, and blocks covered by brick, concrete, metal, and stone. Urbanization and changes in housing, technologies, food markets, and government policies have lessened some seasonal challenges in cities since the mid-nineteenth century.^5^ But urbanization also built heat into the modern metropolis.

Summer in the city has long been hotter than summer in the country. Even in the nineteenth century, the phenomenon now known as the urban heat island (UHI) effect intensified daily highs and extreme heat events in cities. The UHI is a universal characteristic of urban climates created by human activities and the modification of land surfaces.^6^ In the early 1800s, chemist and amateur meteorologist Luke Howard published the earliest report detailing how the orientation and form of London’s buildings and roads led to the absorption and storage of solar radiation.^7^ Howard identified four causes of urban heat storage. First, the geometry of a city can trap solar energy, preventing the radiation that withdraws solar heat from storage in the urban surface. Second, the grouping of structures can create a braking effect that blocks breezes and reduces the amount of heat carried away on the wind. Third, street drainage and sewers dry out cities, leaving less moisture for evaporative cooling. Fourth, anthropogenic sources of heat raise temperatures. Twentieth-century climatologists only identified one additional contributing factor: the thermal properties of urban materials. Urban surfaces have high thermal admittances, which means that they are particularly good at absorbing and holding heat. Landscapes dominated by stone and concrete absorb heat faster and store it in higher quantities than those dominated by plants, soil, and water.^8^ In 1899, one commentator noted how “a stench of hot asphalt and baked brick permeates London, and after hot, sleepless nights come hours of torment for man and beast.”^9^

The UHI magnified the feeling of summer. All three cities experience temperate climates with four seasons. For Paris and London, an oceanic climate generally brings frequent wet weather and mild summers, although Paris is slightly warmer, with summer averages in the high seventies Fahrenheit (mid-twenties Celsius). New York City sits at the boundary of the humid continental and humid subtropical climate zones, with hot, humid summers that average in the low eighties Fahrenheit (high twenties Celsius). All three cities have historically experienced summer highs above 90 °F/32 °C. Key climatic differences between the cities are, however, apparent—most notably New York’s humidity. A day with an air temperature of 90 °F/32.2 °C and 45 percent humidity feels like 93 °F/33.9 °C. But since humidity compounds the feel of heat, the same temperature with 70 percent humidity feels like 105 °F/ 40.5 °C. New Yorkers despised their city’s punishing humidity. As a humorous poem in the *New York Age* summarized in August 1890: “Why do I pant in want of breath/And long e’en for the chill of death/Feeling it must far better be/Than this vile thing—Humidity?”^10^ In a July 1911 heat wave, temperatures peaked at 100 °F/37.8 °C. But high humidity acted as “an agent of almost equal capacity with heat for causing discomfort to humanity”; “[t]he monstrous devil that had pressed New York under his burning thumb for five days could not go without one last curse.”^11^ In London and Paris, high humidity combined with heat much less frequently, and was often associated with thunderstorms at the end of a heatwave rather than a summer-long sensation.

The very form and fabric of cities, combined with summer weather, subjected residents to episodes of extreme heat. We explore how, at the turn of the twentieth century, working-class and poor Parisians, New Yorkers, and Londoners sought relief from summer temperatures that the UHI effect exacerbated. A cold body could be warmed by a stove in the winter, but technology to cool a hot room or body remained elusive. Instead, for most city dwellers, respite from the “horrors of the stifling days” could primarily be found in water.^12^ Across all three cities, baths, pools, shorelines, rivers, fountains, and—in New York, sidewalk fire hydrants—lured overheated urbanites seeking watery coolness.

Yet the modern metropolis could not meet the demand for summer coolness: it lacked sufficient infrastructure and adequate administrative responses. Urbanites were resourceful in their search for thermal comfort and often relied on informal measures that contravened municipal regulations. In Paris, New York, and London, innovative and sometimes joyful working-class geographies of watery coolness emerged when the mercury rose. The sensory dimensions of environmental experiences add a new thermal layer to the maps of cities. Heat’s typical impact on bodies and cityscapes established a collective sensory encounter with summer at the turn of the century. Visual culture and mass-produced newspapers captured (often with dramatic effect) experiences of heat and how residents sought coolness through water. This thermal layer of urban life furthermore reveals that summer weather was unevenly experienced in the modern city. The tactics urbanites used to cool off helped mark social distinctions between rich and poor, adults and children, women and men, and urban and rural life. Summer in the city has a unique sensory history.^13^

In Paris, London, and New York, many urbanites remained trapped within the sensory discomfort of the UHI in summer. One overheated Parisian demanded in 1884: “How can you find a little coolness to fight this fever that burns you, that you breathe in with the air?”^14^ Water was key to thermal comfort and health. As summertime heat crept into and transformed urbanites’ lives, city dwellers set out to make the most of the precious liquid on their journey to coolness. We begin by exploring the inadequate provision of bathing and other water-based cooling opportunities across our three cities and then show how urbanites sought individual and collective sensory relief through informal measures that flirted with illegality. This resourcefulness and flouting of municipal regulations led to bathing in prohibited spots and, in New York, hydrant cracking.

## Unequal Sensory Histories of Urban Heat

Experiences of summer heat are politicized and situational. Class and power relations among different classes structured sensory experiences across Paris, New York, and London’s thermal landscapes. The histories we explore here expose the intertwined thermal and environmental challenges of summer that affected residents in strikingly unequal ways. In revealing these thermal histories, we contribute to an emerging critical heat studies agenda that seeks to move beyond a global, numerical framing of climate change and that challenges the normative emphasis on individual resilience. We take inspiration from the climate justice movement, recognizing that the impacts of a changing climate will not be felt equally. As Zoé Hamstead argues, “social-spatial segregation and built environment-related hazards co-constitute each other.”^15^ Temperatures and the means of thermal regulation are experienced unevenly across social groups and at granular scales (such as by neighborhood), at any given time, and across time. Furthermore, racial segregation and prejudices can limit access to cooling infrastructure, compounding the unequal distribution of thermal comfort created by class, gender, and the built environment.^16^ Questions of climate justice and injustice crystallize in the history of seasonal (dis)comfort in cities. As Hsuan Hsu argues, we are not just experiencing “a single, warming climate but a proliferation of vastly disparate micro-climates.”^17^ When intense summer temperatures became a shared urban experience, heat created new relationships to the city itself, remapping social geographies and provoking classed power struggles over access to water. City dwellers, individually and collectively, responded to the sensory inequalities of urban heat.

Ideas of health framed these responses to discomfort and heat exposure across the late nineteenth and early twentieth centuries. Summer was traditionally a season of ill-health and epidemics in cities globally, bringing epidemics of yellow fever, cholera, and typhus. The wealthy of Philadelphia and New York, for example, fled their cities during summer epidemics in the 1790s and 1830s, respectively.^18^ Since summer weather exacerbated decay, in the wrong environment (like a city), people believed that heat and humidity could potentially produce deadly miasmas.^19^ Yet even as germ theory gained ground in Europe and then the United States and fear of miasmas waned, older ideas about heat and disease remained. In New York, for example, worries about the dog days causing rabies persisted into the early twentieth century.^20^

Class mattered too. When temperatures soared in the summer, wealthier residents had options. They could employ servants to operate punkahs and fans, laze in the shade, or purchase ice for cooling drinks and desserts. Unequal economic, cultural, and social structures shaped perhaps the most obvious responses to urban heat: escape. Well-to-do Parisians escaped to the breezy seaside resorts in Normandy and Brittany. Monied New Yorkers sought out the beaches of Long Island, Rhode Island, and New Jersey, or the woods of the Catskills or Poconos mountains.^21^ Wealthy Londoners decamped for fashionable resorts on the south and east coasts. With the wealthy in cooler climes, those who remained in the cities were exposed to sensory discomfort and real and imagined worries concerning heat and the seasonal cycle of disease.

Across all three cities, all classes of laborers who toiled out-of-doors—construction workers, icemen, deliverymen, and mailmen—suffered in hot weather. Fierce heat also impacted working-class employees indoors. New York physicians worried that workers in industries “where fires are required” were almost as liable to heatstroke “as though they were actively engaged in the midday sun.”^22^ The *New York Tribune* declared, “no class of people probably suffer more from the heat than those obliged to work in factories” like cigar factories, “where the windows are not allowed to be opened on account of the materials manufactured.”^23^ New York’s sugar refineries were also terrible in summer; workers would collapse due to heat.^24^ The laundry and tailoring trades were also punishing. In these clothing industries in London, “whether windows are open or closed, the air becomes vitiated during the heat of the long summer day. Perhaps the presser’s workshop suffers most heavily; windows are shut to keep in the damp atmosphere favorable to the work, and the heat rising from the steaming irons and wet cloth is intolerable.”^25^

Workers’ homes were also hot. C*hambres de bonnes* filled the attic rooms of Parisian buildings built in the late nineteenth and early twentieth centuries. These attic spaces were originally envisioned as empty buffers for the sunlight and heat reaching the zinc or dark-colored roofs, to prevent lower apartments, where the bourgeois classes lived, from overheating. Yet attic rooms were often used as lodgings for servants. In a 1909 report on the living conditions of live-in employees, Paris municipal councilor Émile Massard described the *chambres* as spaces where “Air can rarely flow,” where “There [was] nothing to shield against the heat,” where “Maids […] are stifled under the roofs in the summer.”^26^ Summer temperatures in small New York tenement rooms, where the working-class and poor lived and worked, could top 96 °F/35 °C.^27^

## Taking The Plunge

When homes and workplaces overheated, there were numerous ways to find sensory comfort through water. The need to cool down led to behaviors that digressed from ordinary conduct in an urban setting. Strolling across Paris in 97 °F/36 °C heat, one journalist described the “curious things” that he noticed: a man standing underneath a waterjet in the Palais-Royal gardens, an ice seller consuming his own merchandise, and a florist spraying herself with the water ordinarily used for the flowers.^28^ For a concerned commentator in 1880s London, the oppressive summer heat meant that the “hot weather is serious … the flags are hard, the air is heavy.” Respite from the “horrors of the stifling days” could primarily be found in cool, pure water.^29^ A journalist who broiled during a July heat spell in New York agreed. He averred that “the only relief” in the city “was in water, wherever it could be found.”^30^ There was agreement that water was needed for comfort and health. Yet access to it was marked by inequality despite vast changes in the cities’ watery geographies.

By the 1880s, new water supplies and infrastructure—sewers, drinking water, and, in London and Paris, canals—had ushered in vast changes to urban health and social life. Social reform wrought environmental improvements in all three cities, including housing regulation, street cleaning, waste removal, parks, playgrounds, water fountains, and public baths. Water played a crucial role in nineteenth-century public health advancements grounded in germ theory, which provided a scientific rationale for upper-class cultural dictates around hygiene.^31^ Water also played an important role in the early-twentieth-century drive for physical fitness.^32^ The infrastructure of cleanliness and fitness, most notably baths and swimming pools, doubled as sanctioned spaces for thermal regulation. Modernization and urbanization created the urban heat island, but these processes seldom provided formal ways for urbanites to reduce the strain that city heat exerted on their bodies. Access to pools and baths was a key exception. The product of both private enterprise and municipal endeavor, such spaces were popular forms of infrastructure that urbanites reimagined as sites of summer cooling during periods of extreme heat.^33^ However, repurposing the infrastructure of hygiene and fitness was far from straightforward.

Parisians could bathe in various kinds of public or private, permanent or seasonal, watery infrastructure. This included baths, swimming pools, and the floating summertime “bains de Seine.”^34^ All offered Parisians “a refuge against the heat of the heatwave,” although they differed significantly in their price, cleanliness, location, and size.^35^ Public establishments run by the municipal council were generally more affordable for working-class Parisians, while the cost and clientele of private facilities varied depending on their quality and location, ranging from affordable in working-class neighborhoods to expensive in high-end bourgeois areas.^36^ In the 1890 s, Paris’s municipal council built three public swimming pools (Piscine Rouvet, Piscine Hébert, both in the nineteenth arrondissement, and Piscine Ledru-Rollin in the twelfth) for the health and well-being of urban dwellers.^37^ The Piscine Rouvet was “located in a working-class area … surrounded by coal warehouses, refineries, foundries, mechanical installations of all sorts.” It welcomed “a clientele that [was] as large as it [was] interesting”, no doubt in part because it was free to enter. All three pools were popular among urbanites, and rising temperatures influenced the number of bathers; between 120,000 and 215,000 individuals visited each pool in July and August in the early 1900s.^38^

Parisian pools of all types were popular places during the summer months. They could alleviate the sticky unpleasantness of heat on the body. One journalist rejoiced that “it is so pleasant, when everything around you is cooking, when the asphalt is boiling, when a fiery vapor is rising from the zinc roofs, when toasted leaves are falling from the roasted trees, to stroll under strips of cloth billowing in the breeze, to stroll in light underpants, sheltered from the relentless sun, within reach of a voluptuous, refreshing *dip*.”^39^ The baths attracted “sweaty crowds” in search of relief.^40^ Long queues awaited those who wanted to bathe “on very hot days … crowds of enthusiasts [were] lining up for an hour or more in front of the cold baths, waiting for the great reparative ablution.” And yet, once they reached this “paradise,” “all the cruelties of the wait [were] forgotten.”^41^ But these collective cooling spaces struggled to meet demand and proved ephemeral. Out of the eighteen cold baths registered in Paris in 1889, five remained open in 1921 alongside four public swimming pools.^42^ There were multiple reasons for the closure of the cold baths. They began to be seen as old-fashioned “remnants of another age” in comparison to the newly built permanent swimming pools from the beginning of the twentieth century.^43^ And yet, cold baths remained popular among urbanites. During the heatwave of July 1921, bathing establishments had to turn away customers, as they did not have enough swimming trunks, towels, or changing rooms to meet the demand.^44^

From the middle of the nineteenth century, the allure of the countryside enhanced the appeal of the rivers around Paris for leisure and cooling. By the end of the century, the naturally sandy riverbanks and amenities built for socializing were popular and drew Parisians outside of the city in their search for coolness. At the same time, affordable public transport and increased car ownership gave Parisians, especially wealthier ones, the option to seek coolness further afield.^45^ Yet demand for cooling infrastructure in the summer continued to outstrip supply well into the twentieth century, and it was not until the 1950s that Parisians started to spend their summer outside of the city *en masse*.^46^

Bustling spaces of watery coolness emerged in cities on both sides of the Atlantic. By the end of the nineteenth century, New York beaches lured thousands of residents on summer days and never more so than during a heat wave. City dwellers mobbed the outer-borough beaches on the south shore of Brooklyn and Queens, which offered relatively cleaner waters and large sandy beaches. Some New Yorkers rented bungalows or tents on these shores or along the Upper East River, in the East Bronx, and northern Queens.^47^ The family of play-wright Arthur Miller had one such bungalow and recalled such crowds at Coney Island that it was hard to find a spot to lay a towel on the sand. Beaches could host half a million people during heat waves and holidays.^48^ But as one local journalist took pains to note, although Coney Island was only six miles away from downtown Manhattan, there were “hundreds of thousands in town, bound to the bricks and the flagging like Prometheus to his rock, who find the city hot enough in all conscience.”^49^ Work and other commitments, alongside a lack of resources, meant that not everyone could find relief at the beach.

Baths were a vital way of staying cool within New York’s urban core. While entrepreneurs had long run commercial floating baths, the City opened its first two seasonal municipal baths, open-air floating pools moored at Manhattan piers, in the summer of 1870.^50^ These locations reflected the lack of private bathing facilities in tenements, home to what *Harper’s* magazine deemed the “class of our population who are most in need of thorough periodical ablution—by people who have neither the opportunity to bathe at home nor the means to pay for the use of private bathing institutions.”^51^ Readers knew “the class of population” who lived in tenements and used such baths. As the city’s worst, cheapest housing, tenements traditionally housed immigrants. In 1870, more than 940,000 people lived in New York; 44 percent of this population were foreign-born, and 99 percent were white.^52^ Increased immigration and the spread of tenements went hand in hand. By 1900, over 82,000 tenements housed 2.3 million New Yorkers. Five years earlier, the state legislature mandated that New York’s largest cities run year-round public baths, although years of delay slowed the law’s implementation.^53^ Meanwhile, 97 percent of families living in tenement districts lacked bathrooms.^54^ Defined by crowding, poverty, and ill health, tenements trapped millions of New Yorkers in substandard housing. From *Harper’s* to the writing of Jacob Riis, summer was synonymous with suffering in tenement districts.^55^

Municipal authorities saw public baths as hygienic interventions. Many of the city’s reform initiatives grew out of elite paternalism, dismay with, and revulsion at the dire circumstances in which “the other half lived.”^56^ The city issued time limits and restricted diving and relaxing on swimming platforms, but patrons nevertheless visited for cooling recreation.^57^ While philanthropic baths had opened in the 1890s, New York City only opened its first year-round public bath in 1901. Located at the corner of Rivington and Goerck Streets, it served the overcrowded working-class and immigrant neighborhood of Manhattan’s Lower East Side. The tenements between the Bowery and the East River, which included Rivington, were known as an area of particularly intense suffering during “heated terms.”^58^ Private charity and public indoor baths contained mostly shower stalls and lacked swimming pools. While more utilitarian than the floating baths, locals still turned to public baths to cool off.^59^ Any opportunity to find relief was precious. “The heat of the tenements during the summer months,” one resident declared, was simply “oppressive.”^60^

New York’s municipal and philanthropic bath operators counted visitors, sometimes noting gender and age demographics, but did not record racial demographics. Both types of baths were generally built in tenement districts that housed white, predominately immigrant, communities. At the turn of the century, as baths were opening, patterns of segregation and discrimination hardened, further segregating Black New Yorkers from the white ethnic enclaves most frequently targeted by reformers. It is reasonable to infer that most bathers in tenement district charitable baths and nearby floating baths were white.^61^ While the city’s Black population increased from 13,000 in 1870 to 60,000 by 1900, the city’s largest non-white communities, Black and Chinese residents, comprised only 1.8 percent and 0.02 percent, respectively, of the city’s 3.4 million inhabitants.^62^ The majority of the city’s population, including those who accessed cooling baths in summer, was white.

New York’s floating baths were unsurprisingly most popular in the summertime on very hot days. At the peak of the program in the early 1900s, the Department of Public Works oversaw a fleet of fifteen baths that hosted millions of visitors each summer (men and women used the baths separately). Each bath could fit 100 bathers at a time and hosted 4,500 bathers daily.^63^ The years between 1901 and 1915 were also the heyday for public bath construction; the city built twenty-five new baths (sixteen on Manhattan Island alone).^64^ Both types of baths offered sensory respite from the way humidity collided with New York’s UHI, and their popularity was seasonal: the public baths were significantly underutilized in fall, winter, and spring.^65^ At the baths provided by the New York Association for Improving the Condition of the Poor, bathers set a record for the busiest day on July 22, 1899, and the record for busiest month in July 1900.^66^ One municipal researcher attributed low patronage the rest of the year to the fact that association baths did not include swimming pools and demanded regimented behavior. Reformers had assumed a bathing habit would grow among tenement dwellers if baths were available. Interest, however, emerged seasonally, “through the three or four months of summer. But no such felt want exists to a general degree in cooler weather.”^67^ Tenement dwellers valued water most for thermal relief and gymnastic fun, not necessarily for cleanliness alone.^68^ After 1904, New York City began adding indoor pools to municipal baths, hoping to attract more users. It was not until the 1930s, however, when infamous Park Commissioner Robert Moses built the city’s celebrated outdoor swimming pools and modernized its beaches, that safe and playful summertime swimming became widely available. Moses’s pool network was also notable in that it catered to both Black and white populations, expanding swimming opportunities across the color line.^69^ In the decades before his 1936 pool-building spree, however, New York had but five public pools, and just one outside Manhattan. Baths, not pools, cooled the urban core.^70^

At both New York’s permanent and floating baths, visitor statistics reflected the increasing challenge of summer heat as the season wore on. In June 1911, 7,324 bathers visited the floating baths. July saw an increase to 654,299, and the number of visitors peaked at 812,952 in August. People often queued for access. The number of bathers fell to 334,625 in September and finally, at the swimming season’s end in mid-October, to 9,521.^71^ Clearly a seasonal necessity for thermal regulation, the city’s baths provided a vital—and oversubscribed—collective cooling infrastructure for many New Yorkers. During a hot spell in August 1906, a scuffle over access led to the arrest of Benjamin Silverman of Delancey Street; the court Magistrate who oversaw Silverman’s arraignment declined to hold him, saying he “appreciated how hard it was for one to hold his temper in hot weather.” Nonetheless, the judge fined him $5.00.^72^

In London, meanwhile, inequality and shortages also marked access to water. Wealthier Londoners had long had access to plunge pools, spa waters, and floating baths, which provided opportunities for a cooling dip during periods of summer heat. For example, in the 1870 s, a privately owned and elaborately covered floating bath at Charing Cross apparently delivered filtered, aerated, and cool water and, at twice the cost of other first-class baths, offered an exclusive site for a refreshing dip.^73^ Sensory comfort came at a price. Wider municipal provision was complicated by London’s parochial governance structures, but nonetheless from the 1840 s on, legislation allowed local authorities to build baths and wash houses for the working classes across the capital. Imagined by reformers and authorities as sites of cleanliness and decency, public baths also provided opportunities for escaping summer heat.^74^ In 1888, the new pool at the People’s Palace in Mile End provided respite from hot weather in a neighborhood where a cooling swim was otherwise unavailable or unaffordable to the “decent poor.”^75^ By the 1890 s, there were thirty-four public baths in London, including one on Caledonian Road in Islington (1892) and one on Mansford Street in Bethnal Green (1895).^76^ By 1908, forty-four baths and pools had been built across the city.^77^ These indoor facilities were supplemented by formally sanctioned and free access to bathing and swimming lakes in parks and open spaces across the capital. For one nineteenth century commentator, a long swim in Hyde Park’s Serpentine or Victoria Park’s ponds was “infinitely preferable to any cold or tepid swimming bath.”^78^ By the early twentieth century, Londoners were able to take a cooling dip in eight bathing lakes, including those on Hampstead Heath.^79^

In summer, these facilities were in high demand. Imagined as London’s seaside, the lakes in Victoria Park provided “temporary refuge from the heat.”^80^ One observer enthusiastically estimated that the main lake received twenty to thirty thousand bathers over the course of a hot summer evening.^81^ Whether the estimate was accurate or not, contemporary photographs suggest that it was a very popular place to take to the waters to escape the summer temperatures.^82^ But as attitudes toward hygiene and exercise changed, so did access to such sites of thermal regulation. In the nineteenth century, the lakes in Victoria Park had been sanctioned as bathing ponds, providing an alternative to cooling off in the adjacent canal, but by the twentieth century, high demand and the associated poor water quality saw the ponds replaced by an open-air lido with a modern filtration plant.^83^ The new facility’s large capacity of 1,000 bathers was nonetheless much less than the bathing ponds that it replaced. A tension existed between modernization and meeting the demand for thermal comfort. Reconstruction did make it easier for authorities to count the number of bathers. For example, the three baths owned by the Metropolitan Borough of St Pancras at King Street, Prince of Wales Road, and Whitfield Street attracted 248,069 bathers during the 1910 summer season.^84^ A simple extrapolation of the St Pancras figures across all the indoor public baths in London at the time suggests that there could have been up to 3.5 million visits each summer (out of a city population of around 7 million). With such high demand, the facilities often reached maximum capacity, and prospective bathers had to queue for hours or were turned away altogether.^85^

## Inequitable And Unpleasant

Yet high visitor numbers hide patterns of inequitable access to the cooling properties of purpose-built amenities. Such spaces reflected and reinforced normative assumptions about the social and spatial segregation of public spaces along race, class, and gender lines. As Hsuan Hsu and Eric Dean Wilson have shown, in the United States, racial segregation historically denied Black people safe, dependable, and widespread access to cooling infrastructures, including pools and beaches, as part of what Hsu terms an “effort to reinforce the color line in thermal terms.”^86^ While New York lacked state-sanctioned *de jure* racial segregation, *de facto* practices kept commercial leisure ventures and some public spaces racially segregated across the turn of the century. “No shore” existed, a city real estate expert said, where Black New Yorkers “would possibly be welcome” alongside white patrons.^87^ As a *New York Evening Post* reporter observed, the lack of good recreation facilities was part of the problem of segregation in the city. White New Yorkers protested attempts to open bathing beaches to serve Black patrons; for example, Black real estate entrepreneur Solomon Riley tried to open the city’s first two such venues in the East Bronx in the 1920s. Both ultimately shuttered in the face of racially motivated lawsuits and investigations, and the Great Depression.^88^

In London, municipal facilities were invariably segregated into first-, second-, and sometimes even third-class baths, reflecting and reinforcing the widespread division of leisure, transport, and other infrastructure along class lines in turn-of-the-century Britain.^89^ In Paris, the offerings also catered to various classes, from cheap facilities affordable for laboring populations to high-end establishments for wealthy urbanites. The facilities differed in terms of price, cleanliness, customers, locations, and size.^90^ On one end of the spectrum, the “elegant” pool in the wealthy Monceau area had sterilized water and a manager with “good morals” where young women could learn to swim and enjoy pâtisseries afterwards.^91^ On the other were the municipal pools constructed in the working-class districts and visited by the local laboring populations.^92^

Cooling infrastructure similarly reflected and reinforced gender norms. Of the quarter of a million visits to the St Pancras baths in the summer of 1910, less than a quarter were by women.^93^ Access to the fresh air and cooling water of outdoor ponds was similarly constrained; of London’s eight bathing lakes, five were open only to men, and in the other three, women were able to swim just one day each week.^94^ Although Parisian women enjoyed more opportunities to bathe in the second half of the nineteenth century, most bathers were men.^95^ The Piscine Rouvet was male-only, and the Piscines Hébert and Ledru-Rollin were only open to women one day each week. In the latter two facilities, the loan of a bathing towel and costume cost twenty cents for men and forty cents for women.^96^ The combined effect of restricted and more costly access meant that the number of women visiting municipal swimming pools was low. During the particularly hot summer of 1911, out of the 102,887 people who visited the Piscine Ledru-Rollin, 99,815 were men, and 3,072 were women.^97^ Paris’s private baths were slightly more accessible; some were only open to women, but had a considerably higher entrance fee of sixty cents. In New York, too, there was often very limited entry to such facilities for women, underscoring the highly gendered nature of access to the cooling properties of water in the summer.^98^

For those who were able to access cooling infrastructures, visiting bathing establishments was a vivid sensory experience, one that could combine a complex mixture of welcome coolness and offensive revulsion. In 1896, for example, one Parisian observer described how “you venture shakily along a damp and slimy wooden boardwalk, striving to avoid the puddles of water that stagnate everywhere, rotting the poorly joined planks through which you can see gleaming, beneath your feet, the dirty, yellowish Seine.”^99^ Eventually, “you dive into the yellowish water” of the bath. In doing so, bathers had to reconcile their disgust at entering the filthy water with the cooling properties of a swim. In 1897, another commentator went even further in describing what they had witnessed, writing that

even protected with the carapace of a diver, we would be repulsed to immerse ourselves in this impure sludge, and we wonder by what energetic fumigation those who come out of this pit of manure can be cleansed of the nauseating effluvia of a water that is greasy to the touch and which, if dried by rubbing both hands together, releases a very pungent odor.^100^

One commentator used the oxymoron “fetid freshness” to describe the feeling of Seine water, encapsulating the complex experience of bathing in the river during this period. While the feeling of water on the skin may have helped Parisians cool off, they also had to contend with foreign objects (*corps* *étrangers*) present in the open waters of the Seine, including dead dogs, weeds, and refuse.^101^

In London, a disdainful observer similarly described bathing spots as “dingy, uninviting tanks, situated in dingy uninviting back streets,” full of “unsavoury liquids” that were invariably “something compounded of mud, water, and a stink.”^102^ One New Yorker reflected on his youthful summer days in the early 1900s spent swimming in the East River: “I often marvel that I did not contact typhus as I several times, at low tide when the water was muddy and filthiest, swallowed some of that water which contained the sewerage of several million people.”^103^ In the 1890 s, a floating bath in Brooklyn, anchored near a sewer outlet, caused an eye infection outbreak. Even though New York physicians and sanitation experts repeatedly advised against swimming in harbor waters, and the city began filtering its floating bath-water due to pollution in 1914, public health concerns did not always stop people from taking a dip when a trip to the beach was impossible.^104^ The inequities caused by class, gender, and poverty meant that thermal relief might only just outweigh the tactile, olfactory, and visual unpleasantness of urban waters.

Indoor and outdoor baths and, in New York, beaches, provided one way for city dwellers to adapt to summer temperatures and the UHI effect, often as an unintended consequence of attempts to cleanse the modern city and its people. However, access was constrained by official regulation, social norms, household economics, and inadequate provision. The potentially cooling benefits of water were also dependent upon individual judgments about the sensory compromises involved in using such infrastructure.

## Informal and Illicit

To overcome the thermal inequalities that were baked into the urban environment, poorer city dwellers turned to temporary, informal, and illicit methods to secure the cooling sensation of water. Rivers and canals, fire hydrants, and fountains were reappropriated during the summer to provide fleeting sensory relief from the heat, revealing both the resourcefulness and creativity of city dwellers who were otherwise unable to cool down and the occasional benevolence of city authorities.

The Préfecture de Police de Paris—the authority charged with regulating the use of public spaces in the city—banned bathing in the open waters of the Seine, Marne, and canals in the middle of the nineteenth century.^105^ According to the authorities, the ban was “in the interest of public decency” and to “prevent bathing in dangerous places.”^106^ From the 1870 s, the Préfecture created designated areas of supervised bathing. Every year, bylaws listed the locations where river bathing was permitted. However, those areas were all located on the periphery of Paris, on the Seine, or its eastern tributary, the Marne.^107^ The location and size of these official bathing areas were much criticized. Indeed, they were “too few or too far away for many laborers.”^108^ One journalist declared: “Parisians, who believed you had the right to throw yourselves into the fresh waters of your rivers, think again! You have been granted 275 meters of Seine or Marne; that is really not enough.”^109^

**Figure 1:**
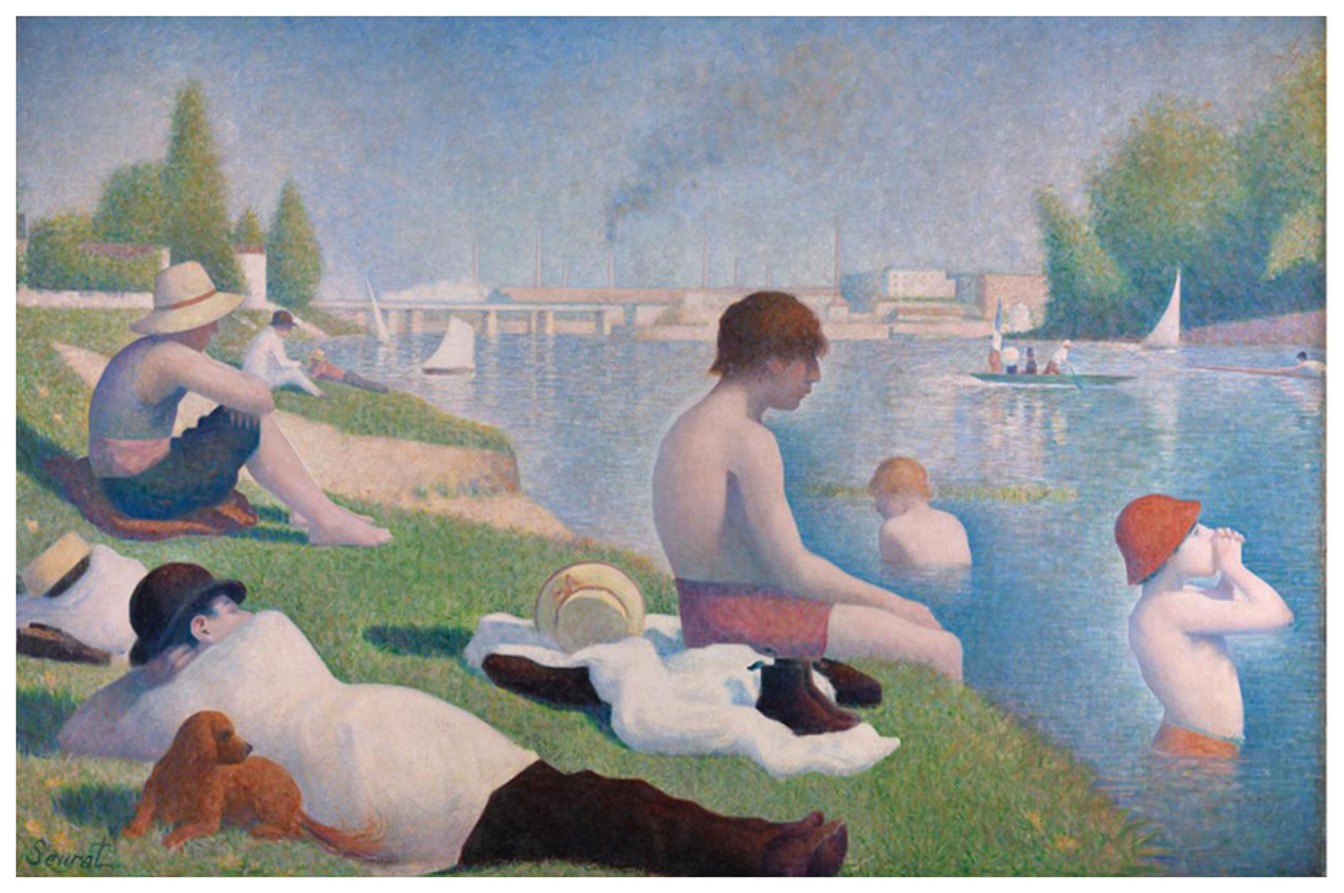
Georges Seurat, Une baignade à Asnières, 1884, oil on canvas, 201 × 300 cm, National Gallery, London. CC BY-NC-ND 4.0.

**Figure 2:**
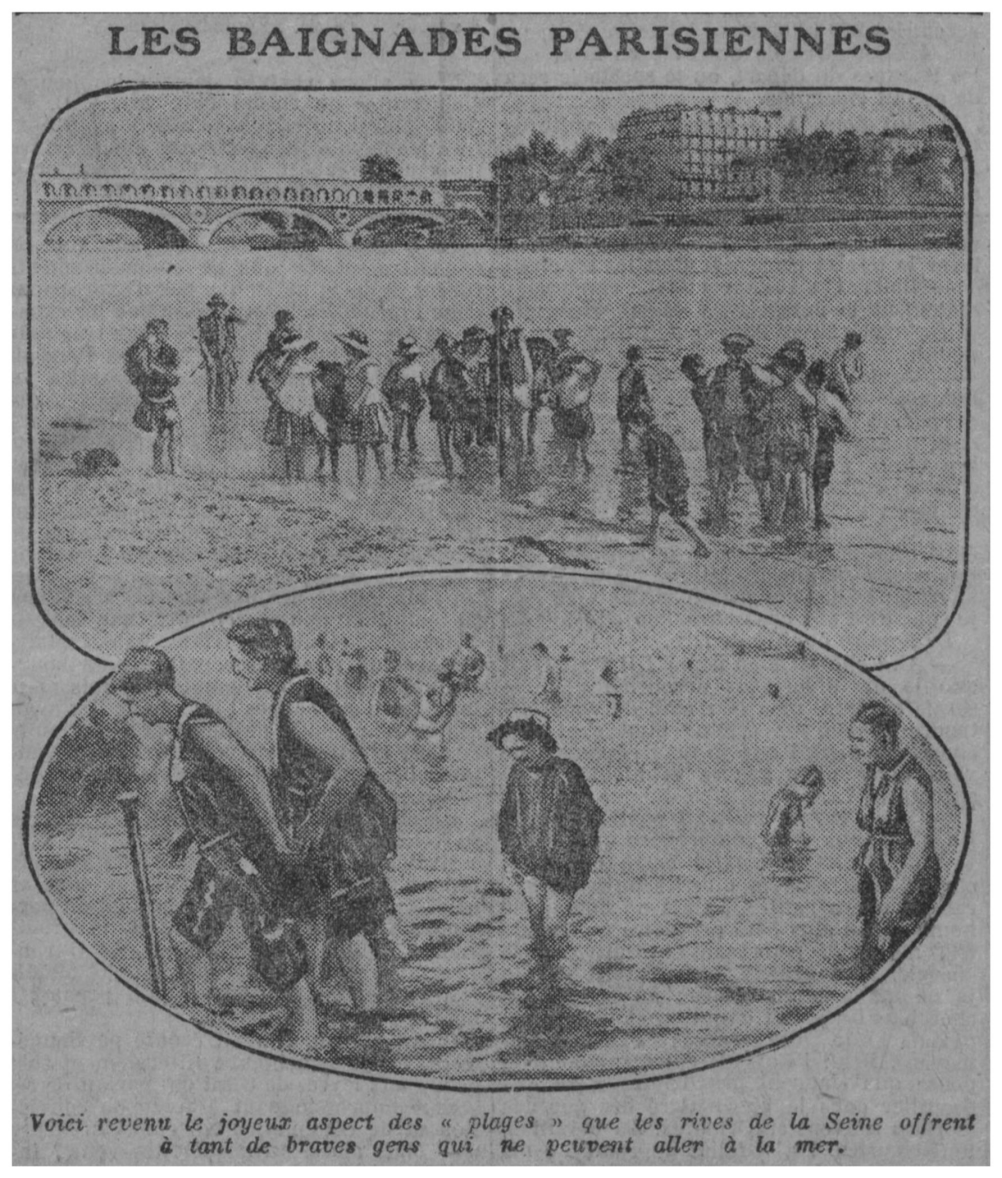
“Les baignades parisiennes,” Le Journal, July 3, 1914, 1. *Source:* gallica.bnf.fr/Bibliothèque nationale de France.

**Figure 3:**
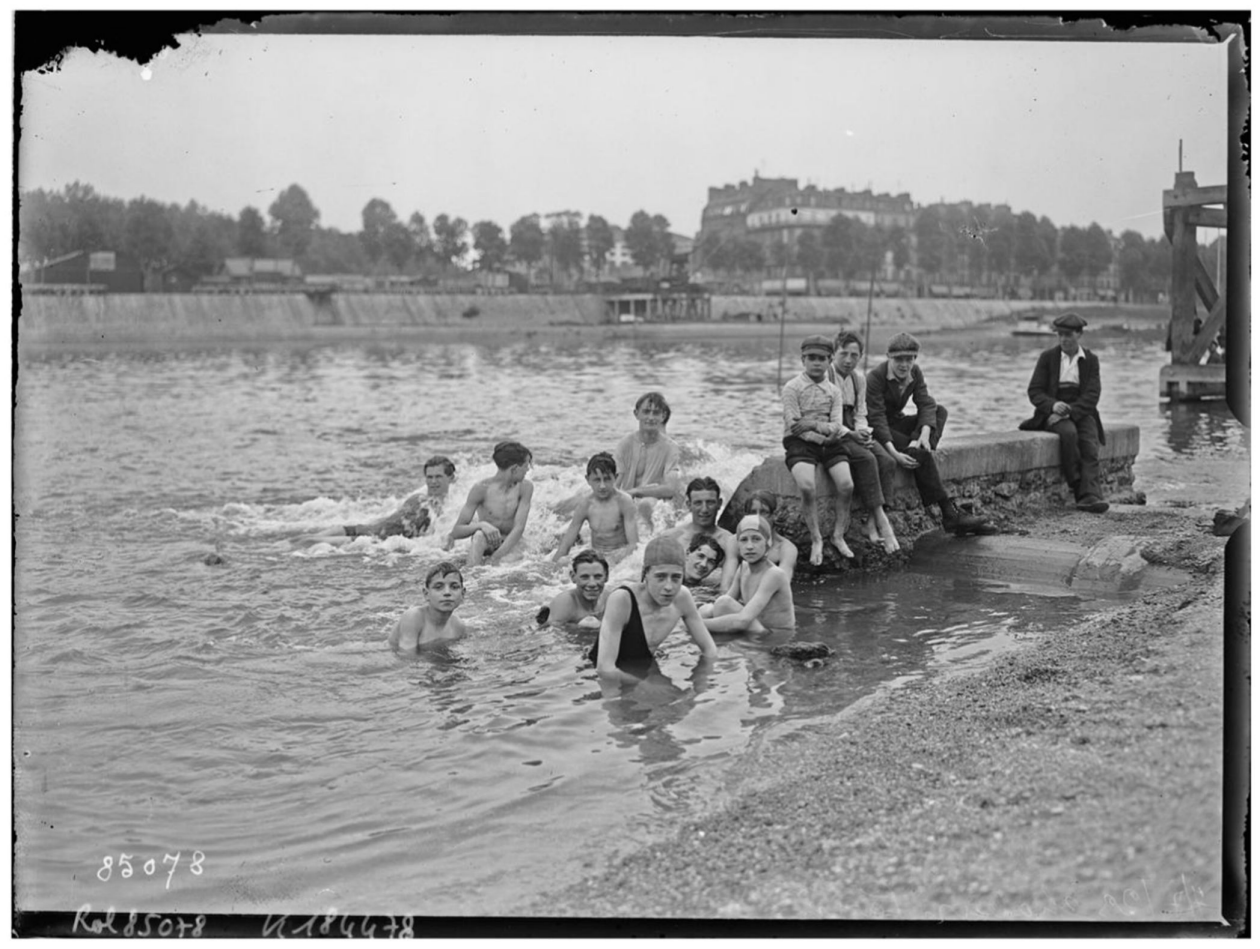
Agence Rol, “7/7/23, chaleur à Paris,” 1923, glass negative, 13 × 18 cm. *Source:* gallica. bnf.fr/Bibliothèque nationale de France.

**Figure 4:**
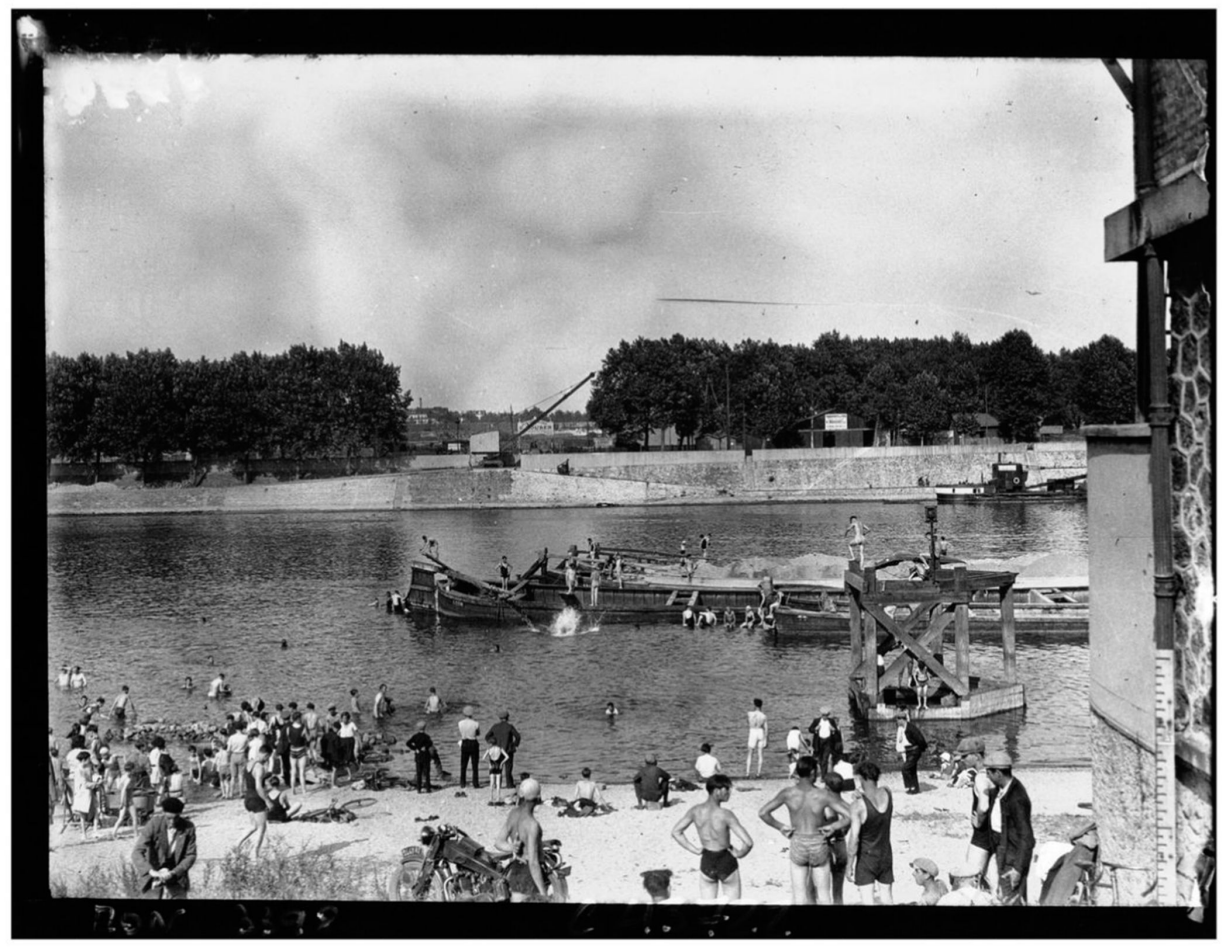
Agence Mondial Photo-Presse, “A Ivry, vague de chaleur: bain libre,” 1932, glass negative, 13 × 18 cm. *Source:* gallica.bnf.fr/Bibliothèque nationale de France.

When baths and pools were unavailable because they were full, unaffordable, or too distant, the Seine offered an alternative, even if bathers risked being fined by policemen on the beat.^110^ In some rare cases, the agents in charge of public safety turned a blind eye, as was the case in the summer of 1899, when bathers were left to enjoy the open waters of the Villette and Bastille neighborhoods for nearly two weeks.^111^ Despite the official ban on river bathing in Paris, some urbanites in search of freshness ventured into the Seine’s open waters in the city center to achieve a degree of thermal regulation. Open-water bathing in Paris was extensively documented by contemporaries, who took to their paintbrushes and later cameras to capture those experiences of respite from summertime heat in the coolness of water. Painter Georges Seurat famously depicted a scene of summertime bathing in the Seine in Asnières, a town on the outskirts of Paris (Figure 1). *Une baignade* *à* *Asnières* (1884) depicts a group of young, seemingly working-class bathers enjoying a quiet riverbank and cool water on a sunny day. And yet, respite from the heat and from the city appears limited. Buildings, chimneys, and smoke—standing in for the ills of modern urban and factory life—are an ominous presence. Although the bathers can access cooling water and leisure, the river in which they swim seems tainted by the pollution escaping from the activity in the background.^112^ This contrast not only heightens the importance of water as a cooling mechanism, regardless of its quality, but also sheds light on the differentiated access to coolness and leisure that class distinctions fostered. The motif of riverine bathing, with visible class undertones, in or near the city persisted into the twentieth century. Although dangerous, the activity was often praised in the press, with journalists describing the “brave folk” who, unable to afford a trip to the seaside, turned the banks of the Seine into beaches (Figure 2). Scenes of urbanites’ enjoyment of the Seine’s waters during heatwaves were extensively photographed. Photos show groups of friends and families in the water, with the omnipresent urban activity and built environment in the background, and point to the key role that rivers continued to play in finding coolness in Paris (Figures 3 and 4).

**Figure 5:**
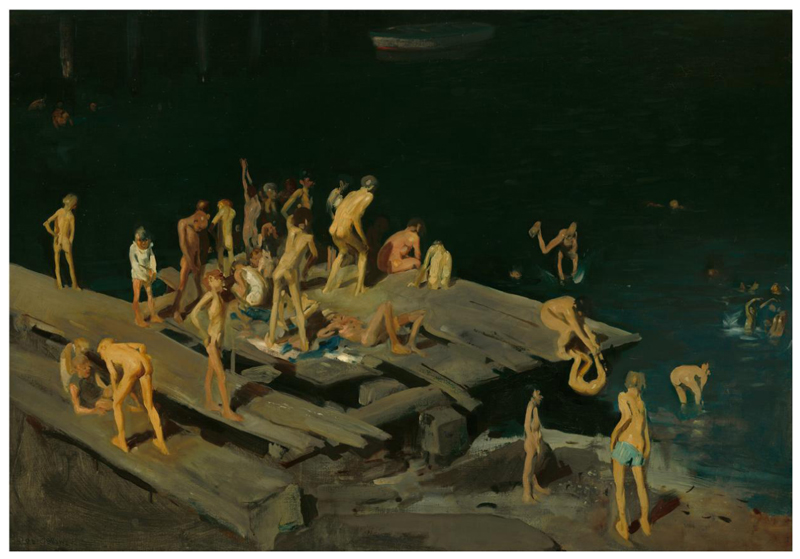
George Bellows, Forty-two Kids (1907), oil on canvas, 42 × 60 1/4 inches, National Gallery of Art, Washington DC. This object’s media is free and in the public domain (more info from the gallery here).

Poor, young boys also braved tides and sewage pollution to swim illegally in New York Harbor. George Bellows immortalized East River swimmers in his 1907 painting *Forty-Two Kids* (Figure 5). Boys watched their peers clamber up pilings and leap from a dilapidated wooden wharf to splash among moored vessels, at risk of having their clothes stolen or being chased by the police for swimming in unsanctioned areas.^113^ Poet Walt Whitman celebrated New York’s youthful swimmers, extolling “The laughter, voices, calls, responses—the springing and diving of the bathers from the great string-piece of the decay’d pier … in to a transparent tea-color—the frequent splash of the playful boys, sousing—the glittering drops sparkling.”^114^ Whitman did not mention, however, the increasing problem of industrial and sewage pollution in New York Harbor, a problem for both Seurat’s Parisian swimmers and Bellows’ New Yorkers. And while youthful swimmers charmed the poet, they were often censured due to class bias. When Bellows used the slang “kids” in his painting title, he referenced gangs of young people associated with hooliganism and working-class immigrant neighborhoods. One art critic found Bellows’ subject “sordid”; another dehumanized the youth of this painting, claiming they resembled “maggots more than humans.”^115^ Middle- and upper-class New Yorkers considered such youth suspiciously, as a social problem, and even reform-minded citizens condemned them as “dock rats” akin to “homeless street boys [and] gutter-snipes.”^116^ Illicit swimming is a window into more than summer days; *Forty-Two Kids* is a window into how heat crystallized class biases and thermal inequalities in the city.

**Figure 6:**
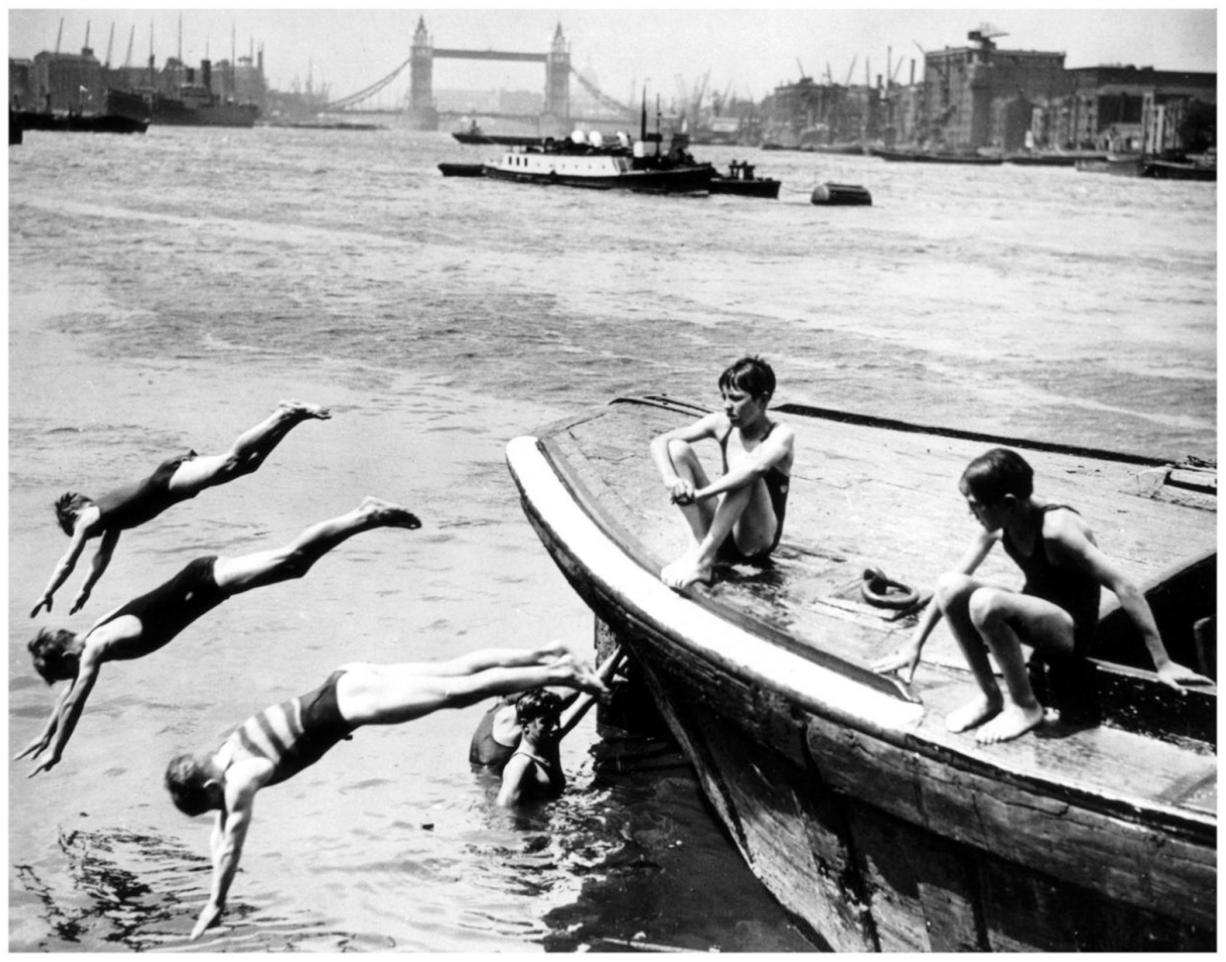
Boys swimming in the River Thames at Rotherhithe, London, 26 July 1934, © Daily Herald Archive courtesy of Science Museum Group. All rights reserved.

Londoners had long sought relief from the summer heat in rivers and canals.^117^ Although smaller rivers, such as the Fleet, had been depicted by artists in the eighteenth century as popular places to cool off, by the late nineteenth century, most had been enclosed, subsumed into the sewerage network, and were unavailable for a cooling dip.^118^ Nonetheless, the city’s main river, the Thames, remained a site of thermal regulation (Figure 6). In his 1885 survey, Charles Dickens Jr. praised the river’s qualities as a place to escape the heat and cool the body: “Few things are pleasanter on a hot day than a plunge into one of the deep, quiet, shady pools in which the Thames abounds.”^119^ However, taking a cooling plunge from unauthorized places on the bank or without appropriate bathing attire could result in a fine from the Thames Conservancy, the organization charged with managing navigation on the river.^120^ Taking a dip in London’s extensive canal network could lead to similar results. Although there are few written accounts of the cooling benefits of bathing in such watercourses, the frequency of newspaper reports covering arrests, fines, and fatalities associated with such behavior, particularly among working-class boys and young men, suggests that a swim was a regular occurrence during the summer heat.^121^ A 1905 photograph of boys bathing in the Grand Union Canal reveals some of the challenges facing urbanites when attempting to reach the glistening water. A tall retaining wall, several meters high, suggests a long drop from the street, while the lack of a towpath means there are few easy access points to get in or out of the water. In the foreground, several boys just manage to keep their heads above the water, presumably out of their depth in the middle of the canal.^122^ Canals were a watery infrastructure primarily intended for transport rather than cooling; contemporary reports described the efforts of canal company employees and the police to limit access to their water. However, the scale of the canal network and its porous boundaries meant that these efforts were invariably unsuccessful; when an illicit bather was escorted to the local police station, it left the coast clear for others to take the plunge.^123^

**Figure 7:**
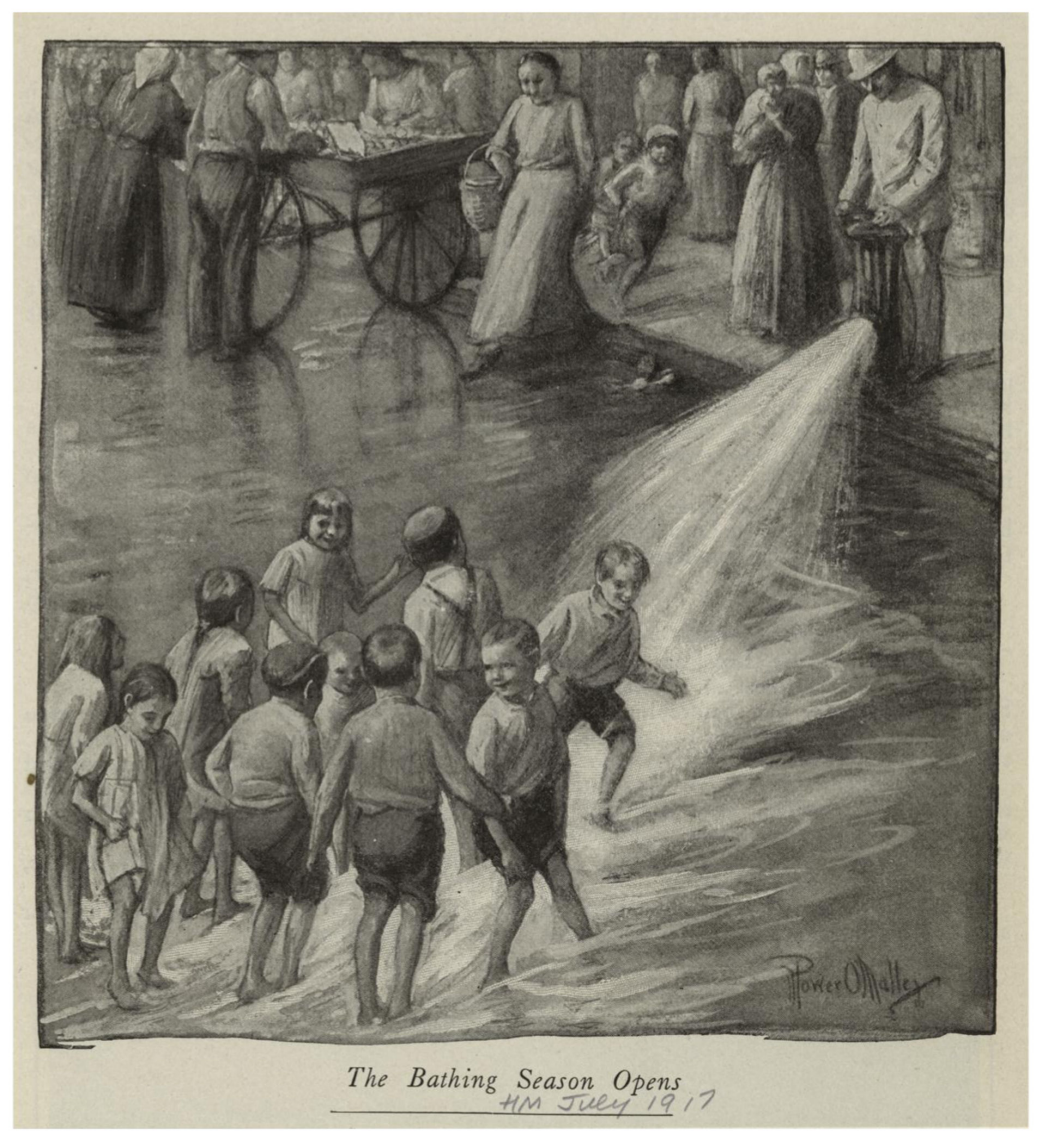
Power O’Malley, The Bathing Season Opens (1917), Wallach Division Picture Collection, The New York Public Library. From NYPL: The copyright and related rights status of this item has been reviewed by The New York Public Library, but we were unable to make a conclusive determination as to the copyright status of the item. You are free to use this Item in any way that is permitted by the copyright and related rights legislation that applies to your use. (Not in Artist Rights Society either)

Rivers and canals were not the only places to seek coolness in the city. In New York, other types of water sources could temporarily transform a neighborhood. City leaders recognized that a city’s stone and brick held heat and sometimes ordered hydrants to be opened to lessen the UHI effect via evaporative cooling. In a heat wave, a mayor might order the Fire Department or Department of Street Cleaning to cool streets and bring down ambient temperatures, as Mayor Hylan did in 1923.^124^ During that sweltering summer, *La Prensa*, a Spanish-language newspaper that catered to the city’s growing Puerto Rican population, noted “the hydrants in the streets were opened yesterday to refresh the environment a bit.”^125^ During the city’s infamous ten-day heat wave in the summer of 1896, residents stepped into the spray of passing street-watering carts, and desperate mothers held overheated infants in hydrant waters released to cool streets.^126^ Artist Power O’Malley captured a similar scene in his sardonically titled 1917 piece “The Bathing Season Opens.” It depicts not the wealthy on their inaugural shore trips of summer, but barefoot children, without swimsuits, playing in hydrant water (Figure 7). A uniformed official, one of the Department of Street Cleaning’s “white wings,” opens the hydrant. People bathing in water intended for street cleaning symbolized the discomfort of summer in the city. The UHI, combined with summer weather creates class-based bodily vulnerability to weather extremes that the government rarely addresses. What responses the city offered tended to be reactive, rather than systematic or proactive.

During heat waves, the Fire Department might rig a sprinkler explicitly for cooling play.^127^ Far more frequently, however, New Yorkers illegally opened hydrants to transform their local environment with water.^128^ Youthful groups sometimes managed to wrench a hydrant open and jam a stick into the nozzle. Water spurted skyward, and the street became an outdoor shower.^129^ In 1933, the *New York Times* claimed that 400 youngsters, identifiable by bathing suits and improvised skimpy attire, protested “against police interference with their heat-relief activities” outside the West 47th Street police station.^130^ An open hydrant can release 1,000 gallons of water or more per minute; the pressure of the stream makes a hydrant difficult to close without the right tools. But the cooling effect of the water was worth the risk of arrest and the threat to domestic water supplies. The gushing fire hydrant has become a trope of the New York working-class summer.

**Figure 8:**
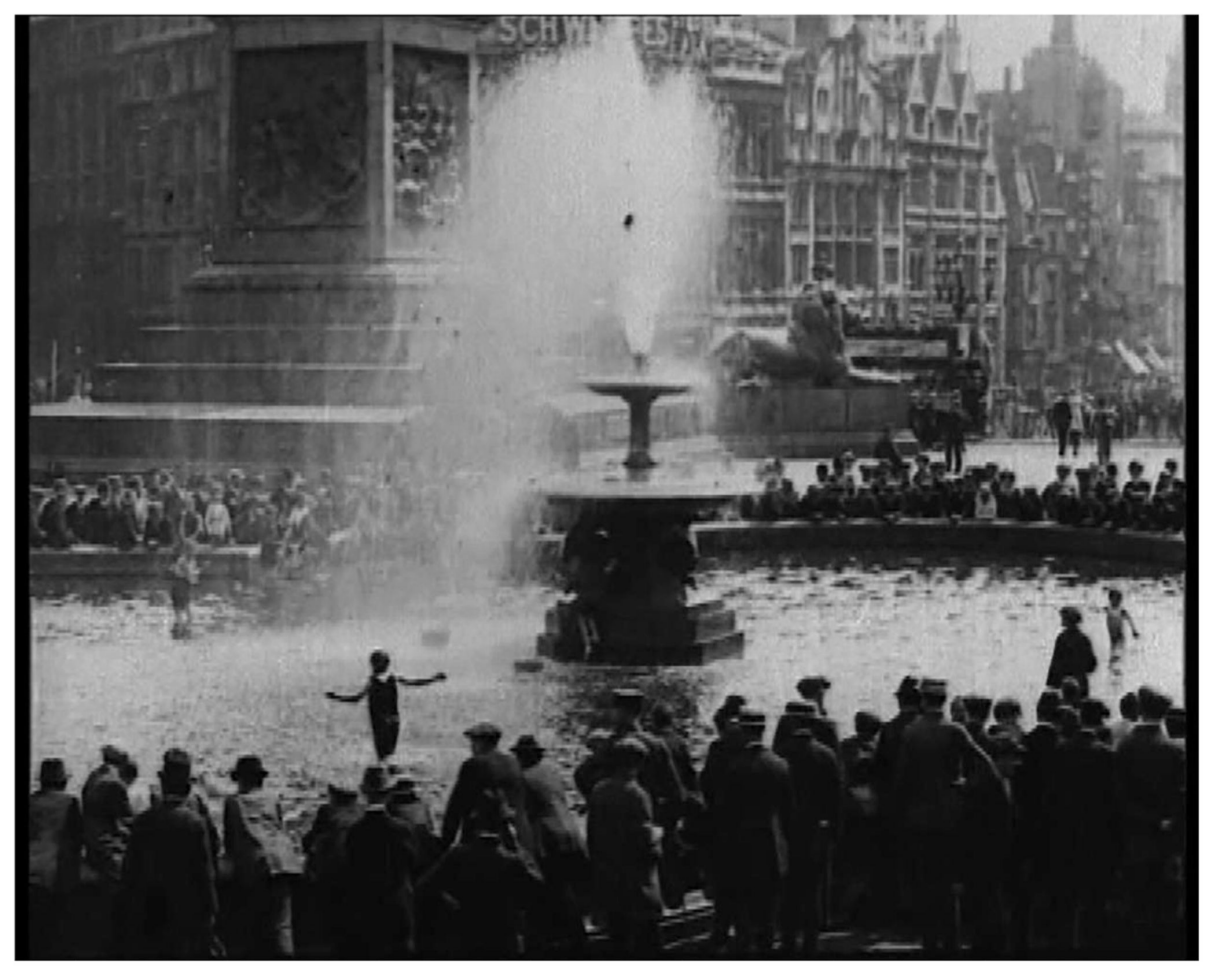
Still from Heat Wave—Trafalgar Square, London, c.1910, British Pathé Archive, 2338.28. All rights reserved.

Urban ornamental water features also provided iconic and illicit opportunities to keep cool. Located close to Fleet Street, the traditional center of London’s newspaper industry, the Trafalgar Square fountains and their basins became journalistic, photographic, and cinematic shorthand for summer in London (Figure 8).^131^ Just the sight of the “towering, splashing water was refreshing” for some Londoners.^132^ For those more intent on submersing themselves, the outcome of their endeavors was far from certain. The police were invariably quick to act when adults attempted to cool off in the fountain basins, even arresting some culprits.^133^ In contrast, officers were often more tolerant of children’s paddling and bathing. For the “poor children to whom the seaside is denied” the water in the fountain basins provided an accessible place to cool off, despite reports of dirty and discolored water.^134^ In August 1923, one newspaper reported that “scores of small children defied the perspiring policemen and paddled happily in the fountain basins of Trafalgar-square.”^135^

Class and age played important roles in shaping who could access the cooling properties of water and where and when this was deemed appropriate by authorities in New York, too. In July 1925, as “the sun rose intolerantly over a baked and soaked city,” children jumped into the fountain in New York’s City Hall Park. Newspaper Row, the industry’s center on Park Row, faced the park, where an ornamental fountain tempted newsboys.^136^ Looking on with a grin, Mayor John Hylan gave the police leave to let the children splash. Alternatingly fanning his face with his hat and mopping his sweaty forehead, Hylan remarked, “That cool water looks good to me. I wish I could join them myself!”^137^ His response, while good-natured, spoke to the ad-hoc character of allowances made to mitigate summer in the city. Such municipal beneficence was not guaranteed—jumping into a fountain could result in legal woes for adults in New York, as in London. Earlier in the summer of 1925, in a June heat wave, theater district dancers jumped into the ornamental pool of the Maine Monument in the center of Columbus Circle. One woman received a court summons.^138^

Swimming in urban waterways could bring more than censure to marginalized communities; it could be a dangerous and sometimes even lethal way to achieve thermal regulation. Non-swimmers or inexperienced ones drowned after striking submerged objects while diving or when overwhelmed, in New York Harbor, by strong currents.^139^ London newspapers routinely reported an increase in drowning during periods of unusually high heat. In August 1876, *The Times* reported ninety-nine deaths from drowning during the preceding seven weeks, while the *Daily Mail* macabrely declared that the summer of 1899 was “the drowning season.”^140^ The latter reported on boys and young men, primarily from working-class neighborhoods, who were carried away by powerful river currents, drowned in the wake of passing ships, or unable to haul themselves out of the deep water around docks and piers. Canals were hardly safer. In August 1913, for example, the *Daily Telegraph* reported on the case of six-year-old Isaac Monish, who drowned while saving a struggling friend who had taken a cooling dip in the Regent’s Canal.^141^ A year later, one reporter gloomily noted that “the canal takes a heavy toll in young lives.”^142^ The creative repurposing of existing infrastructure may have helped Londoners secure a degree of comfort in the face of torrid temperatures, but it was not without significant challenges and risks.

In Paris, too, bodies of water were sites of tragedy. In the early twentieth century, drownings made up a third of accidental deaths in France and occurred almost solely in the summer.^143^ Between 1895 and 1913, the Paris morgue received an average of 295 people each year who died by drowning.^144^ The police recorded some of these incidents. In July 1908, they opened an inquiry into the death of Eugéne Lepelletier, who drowned in Sylvain Arnould’s river bath in Joinville-le-Pont. The next year, in August 1909, Camille Léonard “had wanted to bathe immediately after eating and sank [like a stone].” In August 1910, Paul Delpech, described as “timorous” because he was not able to swim, was led into the water of the river bath “Banc de Sable” by his cousin. There, he lost his footing and tried holding onto another bather’s leg who, thinking he was the target of a prank, shook him away. Upon seeing this, the lifeguard jumped in the water to intervene, but Delpech drifted away in the confusion. His body was only found forty-five minutes after the incident took place.^145^ Concern for the safety of individuals had been one of the original drivers for the Préfecture de Police to ban bathing in the Seine. Despite ordinances banning harbor swimming in New York, summertime drownings remained a concern into the 1930s. Near the decade’s end, Park Commissioner Robert Moses launched a free swim lesson program for both young people and adults to address the city’s 400 annual drownings.^146^ As more people were taught to swim in the twentieth century, fewer people drowned as they tried to keep cool.

## Conclusion

In Paris, New York, and London, heat was an unseen element of the urban environment, but it was felt intensely within the body—the feeling of hot air in the lungs as well as on the skin. The high humidity of New York intensified the thermal discomfort of the UHI. Due to its porous nature and highly sensory faculty, the skin was central to both attempts to cool off and the experience of bathing. Descriptions of the skin encountering water were often made in vivid sensory terms, invariably reflecting a compromise between the feelings of pleasure and disgust that the water and its associated facilities could arouse. Keeping cool in the modern melting metropolis through the refreshing properties of water was an individual and collective challenge. It necessitated resourcefulness, bravery, and a willingness to break rules (whether cracking a hydrant or swimming in prohibited places).

An innovative, and sometimes joyful, working-class geography of watery coolness emerged each summer as the mercury rose. There were similar water activities occurring across London, New York, and Paris. Most notably, all three cities lacked sufficient and inclusive access to swimming pools, public baths, and sanctioned bathing places to cool all residents. Heat and climatic conditions, more generally, were experienced on multiple scales. Water-cooled individual bodies with individual preferences. But the search for and experience of coolness was also often communal, as people swam together in pools and rivers and shared the water that gushed from hydrants. The unintended and informal uses of fire hydrants, ornamental fountains, and commercial waterways to cool off illustrate how urbanites adapted infrastructure to their own purposes. In aggregate, these decisions reveal a social history of cooling off.

Our focus on the experiential and the everyday complements and builds on research into more recent extreme heatwaves, and shows how the search for cool has long been an everyday sensory practice in the modern urban heat island.^147^ Heat is socially produced in specific built environments and eras. By the late nineteenth century, urbanites knew that summer, combined with what we today call the UHI effect, fostered new, city-specific sensory effects. As *Harper’s* magazine observed, big cities, like Paris, London, and New York, were “pretty bad places in midsummer. The bigger they are, and therefore the better as cities, the more intolerable they become in July and August to folks who cannot leave them.”^148^ Yet city leaders and reformers seldom responded adequately or inclusively to the challenge of summer heat. Access to the cooling properties of water was shaped by inadequate municipal provision, inequitable gender norms, economic inequality, individual sensory perception, and attitudes toward risk. Coolness did not come easily or equally, and not everyone was able to secure it. Urbanites were forced to be resourceful, adapting to daily summer highs and punishing extreme heat episodes with behaviors that bridged both the legal and illicit. In all three cities, collective responses established urban vernaculars for keeping cool.

The effort needed to keep cool has persisted. Even as cooling strategies shifted from water to air conditioning in the twentieth century, there remained a sense that hot weather was to be largely endured by individuals. The use of air conditioning increased in commercial buildings in New York in the 1920s and in London and Paris beginning in the 1930s. Climate-controlled theatres, department stores, and hotels beckoned the overheated, reconfiguring thermal experiences of summer. But the air conditioning revolution was far from complete, and the core ways to cool down remained very similar to those of the late nineteenth and early twentieth centuries. Urbanites continue to seek out water to stay cool. Parisians today bathe in the now officially clean Seine, with the 2024 Summer Olympic Games injecting money and publicity into river swimming.^149^ The New York City Department of Parks and Recreation, meanwhile, publishes a map showing where New Yorkers can cool off in water at beaches, pools, and playgrounds where sprinklers keep children refreshed and content.^150^ Twenty-first century Londoners can enjoy lidos, whose financial stability is far from guaranteed, while developers dream of a return to floating baths on the Thames.^151^ Urbanites today face shared environmental challenges in summer due to seasonal hot weather, the urban heat island effect, and hotter, more frequent, and longer heat waves caused by climate change. To the extent that there will be solutions to summertime extreme heat, they will not be purely technological but local and collective, based on a city’s weather, political will, community needs—and inclusive access to the cooling properties of water.

## Footnotes

1 “La Canicule,” *Le Monde Illustr*é, August 23, 1884.

2 Julian Ralph, “A Hot Night in New York,” *Harper’s*, August 17, 1889.

3 “The Heat in London,” *Daily Telegraph*, July 20, 1899.

4 Christopher E. Forth and Elinor Accampo, eds, *Confronting Modernity in Fin-de-Si*è*cle France: Bodies, Minds, Gender* (Basingstoke, 2010); Richard Dennis, *Cities in Modernity: Representations and Productions of Metropolitan Space, 1840-1930* (Cambridge, 2008); David Harvey, *Paris: Capital of Modernity* (London, 2003); Max Page, *The Creative Destruction of Manhattan* (Chicago, 1999). On comparative urban history, across these three cities and beyond, see Nicholas Daly, *The Demographic Imagination and the Nineteenth-Century City: Paris, London, New York* (Cambridge, 2015); Nicolas Kenny and Rebecca Madgin, eds, *Cities Beyond Borders: Comparative and Transnational Approaches to Urban History* (London, 2016); Pierre-Yves Saunier and Shane Ewen, eds, *Another Global City: Historical Explorations into the Transnational Municipal Moment, 1850-2000* (Basingstoke, 2008); Derek B. Scott, *Sounds of the Metropolis: The 19*^*th*^ *Century Popular Music Revolution in London, New York, Paris and Vienna* (Oxford, 2008).

5 Lord Mayor of London, “Street Watering during Drought” (1800), ACC/2558/MW/C/15/374/002, The London Metropolitan Archives; “Our Amazing Summer,” *The Observer*, August 13, 1911. See also the *Journal of Urban History* special section, introduced by: Kara Murphy Schlichting and Avi Sharma, “Urban Seasonality: New Paths in Urban Environmental History,” *Journal of Urban History* 51, no. 1 (2025), 3-9.

6 Gerald Mills, “Urban climatology: History, status and prospects,” *Urban Climate* 10 (2014), 479-489; Gerald Mills, “Luke Howard and the Climate of London,” Weather 63, no. 6 (June 2008), 153-57. For the history of urban heat island research see Iain D. Stewart, “Why should urban heat island researchers study history?” *Urban Climate* 30 (2019), 1-25. Emilien Renou observed the urban heat island in Paris in the mid-nineteenth century. German researchers produced a number of key studies at the turn of the twentieth century, including Julius Hann who coined the German term Stadttemperatur (city temperature) in 1897 and Albert Peppler, who coined the “urban heat island” (Wärmeinsel) in 1929, over 100 years after the phenomenon was first observed. See Stewart (2019), 6, 11. Stewart points out that the concept has been incorrectly credited to mid-twentieth century studies of Great Britain. See Stewart (2019), 11. Many of the temporal characteristics of the urban effect on air temperature that Howard observed have since been confirmed.

7 Luke Howard, *The Climate of London: Deduced from Meteorological Observations, Made at Different Places in the Neighbourhood of the Metropolis*, vol. 1 (London, 1818); Luke Howard, *The Climate of London, Deduced from Meteorological Observations at Different Places in the Neighourhood of the Metropolis*, vol. 2 (London, 1820); Helmet E. Landsberg, *The Urban Climate* (New York, 1981); Mills, “Luke Howard and the Climate of London”.

8 William P. Lowry, “The Climate of Cities,” *Scientific American* 217, no. 2 (1967): 16.

9 “Sun-Smitten,” *Daily Mail*, July 22, 1899.

10 L.H.L., “Humidity,” *New York Age*, August 16, 1890, 2.

11 For “an agent,” see “Heat’s Work Unabated,” *New York Daily Tribune*, July 7, 1911, 2. For “monstrous devil,” see “Heat Wave Ebbs Before Breezes,” *New York Daily Tribune*, July 8, 1911, 8. There are several indices of the overall effect of climate on the body. The most recognized ones are wet bulb globe temperature and the newer universal thermal climate index. Both take in consideration four climatological factors: air temperature, relative humidity, radiant heat, and air movement. See Krzysztof Błażejczyk et al., “An Introduction to the Universal Thermal Climate Index (UTCI),” *Geographic Polonica*, 86:1 (2013), 5-10; Sookuh Park, Stanton E. Tuller, and Myunghee Jo, “Application of Universal Thermal Climate Index (UTCI) for Microclimatic Analysis in Urban Thermal Environments,” *Landscape and Urban Planning* 125 (May 2014), 146-155. For a nineteenth-century recognition of humidity and wet bulb temperature see W. F. R. Phillips, “Sensible Temperature,” *Transactions of the American Climatological Association* 12 (1896), 16-25.

12 “Lord Rosebery’s Splendid Gift,” *Daily Telegraph*, May 15, 1888.

13 The scholarship of smell is representative of the ways in which sensory perceptions are historically contingent. See Alain Corbin, *The Foul and the Fragrant: Odor and the French Social Imagination*, trans. M.L. Kochan, R Porter, and C Prendergast (Cambridge MA, 1986); Dolly Jørgensen, “The Medieval Sense of Smell, Stench and Sanitation,” in *Les Cinq Sense de La Ville Du Moyen Âge à Nos Jours*, ed. Ulrike Krampl, Robert Beck, and Emmanuelle Retaillaud-Bajac (Tours, 2013), 301–13; for a recent conversation on the evolution and possibilities of sensory scholarship, particularly smell, see William Tullett et al., “Smell, History, and Heritage,” *The American Historical Review* 127, no. 1 (2022): 261–309.

14 “La Chaleur à Paris,” *La Patrie*, September 5, 1884.

15 Zoé A. Hamstead, “Critical Heat Studies: Deconstructing Heat Studies for Climate Justice,” *Planning Theory & Practice* 24, no. 2 (March 2023): 153–72, https://doi.org/10.1080/14649357.2023.2201604.

16 Jeff Wiltse, *Contested Waters: A Social History of Swimming Pools in America* (Chapel Hill, 2007); Victoria W. Wolcott, *Race, Riots, and Roller Coasters: The Struggle over Segregated Recreation in America* (Philadelphia, 2012); Andrew W. Kahrl, *The Land Was Ours: African American Beaches from Jim Crow to the Sunbelt South* (Cambridge MA, 2012); Kara Murphy Schlichting, *New York Recentered: Building the Metropolis from the Shore* (Chicago, 2019), 80–119; Elsa Devienne, *Sand Rush: The Revival of the Beach in Twentieth-Century Los Angeles*, trans. Troy J. Tice (Oxford, 2024).

17 Hsuan L Hsu, “Race, Urban Heat, and the Aesthetics of Thermoception,” *American Literary History* 35, no. 2 (2023): 769–94.

18 For scholarship on the seasonal threat of disease in the context of U.S. cities, see J.H. Powell, *Bring Out Your Dead: The Great Plague of Yellow Fever in Philadelphia in 1793* (1949) (Philadelphia, 2014); Charles Rosenberg, *The Cholera Years: The United States in 1832, 1849, and 1866* (Chicago, 1962); and Kathryn Olivarius’s *Necropolis: Disease, Power, and Capitalism in the Cotton Kingdom* (Cambridge MA, 2022). For a global comparative approach see Jim Downs, *Maladies of Empire: How Colonialism, Slavery, and War Transformed Medicine* (Cambridge MA, 2021). On the history of linking climate to illness, see Sabine Barles, “La climopathologie aux XVIIIe et XIXe siécles,” *Les Annales de la recherche urbaine* 61 (1993): 19-26.

19 For example, New York City’s Board of Health blamed high mortality in summer 1868 on hot, humid weather that had cooperated “with local causes of atmospheric impurity…[to] produce excessive mortality among infants.” *Third Annual Report of the Metropolitan Board of Health 1868*, 523. The scholarship on miasmatic theory in public health is vast. For public health and miasmatic etiology in New York in particular, see John Duffy’s classic works *A History of Public Health in New York City 1625-1866* (New York, 1968) and *The Sanitarians: A History of American Public Health* (Champaign, 1992). On London, see Stephen Halliday, “Death and Miasma in Victorian London: An Obstinate Belief,” *BMJ* 323 (December 2001): 1469-1471; and Tom Koch, *Disease Maps: Epidemics on the Ground* (Chicago, 2011). On germ theory in France, see Steven Zdatny, *A History of Hygiene in Modern France: The Threshold of Disgust* (London, 2024).

20 Chris Pearson, “The Sinister Side of Summer: Revisiting the Dog Days of Nineteenth-Century New York,” *Journal of Urban History* 51, no. 1 (2025): 10-20.

21 “Heat Refugees Sail Today on 8 Vessels,” *New York Times*, June 9, 1923.

22 See, for example, Gouverneur M. Smith, “On Sunstroke Delivered at the New York Hospital, N. Y,” *Medical Record*, July 1, 1869, 221.

23 “Fierce Summer Heat,” *New-York Tribune*, July 11, 1880, 12.

24 “Another Day of Misery,” *New York Times*, July 30, 1892, 1; “Sixteen Deaths,” *The World*, July 29, 1892, 5; “Man Boiled to Death,” *New York Tribune*, July 20, 1905, 1.

25 Barbara Drake, “The West End Tailoring Trade,” in *Seasonal Trades*, ed. Sidney Webb and Arnold Freeman (Constable, 1912), 86.

26 Émile Massard, “Proposition relative à l’hygiéne des habitations de Paris en général, et en particulier des logements de concierges, gens de maison et employés logés,” Conseil municipal de Paris (1909), 5, DB/438, Archives de la Préfecture de Police de Paris.

27 Annie Marion MacLean, “The Sweat-Shop in Summer,” *American Journal of Sociology* 9, no. 3 (1903), 291-292, 296.

28 A.S., “Paris vu Par 36°à l’ombre,” *Le Populaire*, July 18, 1928.

29 “Lord Rosebery’s Splendid Gift.”

30 “Nation’s Death List 300,” *New York Times*, June 7, 1925.

31 Coline Lorang, “Les Fontaines Wallace (1872-2012): Hygiène, Esthétique et Patrimoine,” *Soci*é*t*é*s & Repr*é*sentations*, 34, no. 2 (2012): 213–27; Marilyn T. Williams, *Washing “the Great Unwashed”: Public Baths in Urban America, 1840-1920* (Columbus, 1991); John Duffy, *The Sanitarians: A History of American Public Health* (Champaign, 1992); Nancy Tomes, *Gospel of Germs: Men, Women, and the Microbe in American Life* (Cambridge MA, 1998); Mike Wallace, *Greater Gotham: A History of New York City from 1898 to 1919* (Oxford, 2017); Dorothy Porter, *Health, Civilization and the State: A History of Public Health from Ancient to Modern Times* (Abingdon, 1999).

32 Ina Zweiniger-Bargielowska, *Managing the Body: Beauty, Health, and Fitness in Britain 1880-1939* (Oxford, 2010); Susan Currell, *The March of Spare Time: The Problem and Promise of Leisure in the Great Depression* (Philadelphia, 2010); Wiltse, *Contested Waters*.

33 Sun-Young Park, *Ideals of the Body: Architecture, Urbanism, and Hygiene in Postrevolutionary Paris* (Pittsburgh, 2018), see especially Chapter 5, “The Sportsman: Shaping Bourgeois Bodies in Urban Recreational Grounds.”

34 Préfecture de Police, “État des bains froids autorisés en 1894. Arrêté du 18 juin 1894” (1894), DB/227, Archives de la Préfecture de Police de Paris.

35 J. de Rougé, “Chronique du dimanche. Les bains de Seine,” *La Lanterne*, June 4, 1894 “Le bain des Parisiens. D’une piscine à l’autre,” *L’Avenir*, August 31, 1932.

36 Lucie Nicolas, “Longueurs à la piscine. Temps aménagés et temps vacants dans les bassins parisiens (années 1880-1930),” *Revue d’histoire culturelle [en Ligne]* 3 (2021), https://doi.org/10.4000/rhc.705.

37 On swimming pools through the socialist impulse of the interwar years, see Christelle Inizan, “La piscine de Pantin (1935-1937), une réalisation architecturale et sociale dénvergure,” *Livraisons de l’histoire de l’architecture* 14 (2007): 39–53.

38 M.G. Lemarchand, “Rapport—Au nom de la 6^e^ commission (1): 1° Sur le fonctionnement des piscines et ‘etablissements balnéaires municipaux; 2° Sur la création de nouvelles piscines et ‘etablissements de bains-douches (1909),” DB/227, Archives de la Préfecture de Police de Paris; Préfecture de la Seine, *Annuaire statistique de la ville de Paris, année 1900* (1902); *Annuaire statistique de la ville de Paris, année 1910* (1912); *Annuaire statistique de la ville de Paris, année 1911* (1913); *Annuaire statistique de la ville de Paris, ann*é*es 1921 et 1922* (1925).

39 “Les bains froids,” *Le Rappel*, August 20, 1898.

40 “Bains de Seine,” *La République Française*, July 14, 1921.

41 Ernest Laut, “La semaine. Les bains froids. Écoles de natation en Seine. La baignade parlementaire,” *Le Petit Journal Illustr*é, August 12, 1923.

42 “Les chaleurs caniculaires ont-elles une répercussion sur la Seine?,” *Le Petit Parisien*, August 31, 1921; Roger Valbelle, “Par jour Paris consomme actuellement plus de 7 millions de kilos de glace,” *Excelsior*, July 13, 1921.

43 “Maisons flottantes dans Paris,” *Le Journal*, April 27, 1903.

44 Valbelle, “Par jour Paris consomme actuellement plus de 7 millions de kilos de glace.”

45 Isabelle Duhaut, “Les baignades en rivière d’Île-de-France, des premiers aménagements à la piscine parisienne Joséphine-Baker,” *Livraisons de l’histoire de l’architecture* 14 (2007): 9–38.

46 Ellen Furlough, “Making Mass Vacations: Tourism and Consumer Culture in France, 1930s to 1970s,” *Comparative Studies in Society and History* 40, no. 2 (1998): 247–86.

47 “Crowds Fled from Heat,” *New York Times* (Jul. 22, 1895), 3. On the range of beaches in the five boroughs, see Schlichting, *New York Recentered*. On specific Long Island beaches, see John F. Kasson, *Amusing the Million: Coney Island at the Turn of the Century* (New York, 1978) and Lawrence Kaplan and Carol P. Kaplan, *Between Ocean and City: The Transformation of Rockaway, New York* (New York, 2003).

48 Arthur Miller, “Before Air Conditioning,” *The New Yorker* (June 14, 1998), accessed July 3, 2025, https://www.newyorker.com/magazine/1998/06/22/before-air-conditioning. On beach crowds, see, for a typical example, “Heat Wave Grips Most of Nation,” and “Crowds Flee Heat and Sleep in Parks,” *New York Times* (June 7, 1925), 2.

49 Ralph, “A Hot Night in New York.”

50 Mayor’s Committee of New York City, *Report on Public Baths and Public Comfort Stations: Being a Supplementary Report to the Inquiries into the Tenement House Question in the City of New York, Pursuant to Chapter 479 of the Laws of 1894* (Albany, 1897); New York City (Borough of Manhattan), *Public Baths Under the Supervision of the President of the Borough of Manhattan* (New York, 1912); Mars Plater, “Escaping Gotham: Working People and the Nineteenth-Century Struggle over Urban Nature,” (PhD diss, Rutgers University, 2021); Naomi Adiv, “Rivers, Filth And Heat: Riverbaths And The Fight Over Public Bathing,” The Gotham Center for New York City History, May 1, 2018, accessed July 3 2025, https://www.gothamcenter.org/blog/rivers-filth-and-heat-riverbaths-and-the-fight-over-public-bathing; Ann L. Buttenwieser, *The Floating Pool Lady: A Quest to Bring a Public Pool to New York City’s Waterfront* (Ithaca, 2021), 29-44.

51 “Waiting to Bathe,” *Harper’s Illustrated*, July 16, 1870.

52 Mariana Griswold Van Renseelaer, “People in New York,” *Century* (Feb. 1895), 546; Daniel Van Pelt, *Leslie’s History of the Greater New York*, vol. 1 (New York, 1898), 545, and DeForest, *Tenement House Problem*, 194-195.

53 Williams, “New York City’s Public Baths.”

54 “Waiting to Bathe,” *Annual Report of the Department of Public Works of the City of New York for the Year Ending April 10, 1872* (New York, 1872), 36; Wallace, *Greater Gotham*, 549.

55 Jacob Riis, *How the Other Half Lives: Studies Among the Tenements of New York* (New York, 1890), 44, 166.

56 On paternalism and voyeurism among 19^th^ century urban reformers, see Alan Mayne, *The Imagined Slum: Newspaper Representation in Three Cities 1870–1914* (Leicester, 1993).

57 T.M.B. Cross, “Supplement No. 6: Report on Existing Baths in New York,” in *Report of the Tenement House Committee as Authorized by Chapter 479 of the Laws of 1894* (Albany, 1895), 188–203; New York City Borough of Manhattan Public Works Department, *Statistics Relating to Public Baths and Comfort Stations Under the Supervision of the President of the Borough of Manhattan* (New York, 1907), 9; Marilyn T. Williams, “New York City’s Public Baths: A Case Study in Urban Progressive Reform,” *Journal of Urban History* 7, no. 1 (1980): 54.

58 Daniel Van Pelt, *Leslie’s History of the Greater New York*, vol. 1 (New York, 1898), 545.

59 On both private philanthropic and public baths, see Cross, “Supplement No. 6”; Frank Tucker, “Public Baths,” in *The Tenement House Problem, Including the Report of the New York State Tenement House Commission of 1900*, ed. Robert W. DeForest and Lawrence Veiller, vol. 2 (New York, 1903), 33–55; New York City Borough of Manhattan Public Works Department, *Statistics Relating to Public Baths and Comfort Stations*; New York City (Borough of Manhattan), *Public Baths Under the Supervision of the President of the Borough of Manhattan*, 18; Edwin G. Burrows and Mike Wallace, *Gotham: A History of New York City to 1898* (Oxford, 1999), 1198; Wallace, *Greater Gotham*, 550–51.

60 Herman M. Biggs, “Tuberculosis and the Tenement House Problem,” in *The Tenement House Problem*, vol. I, 447; “The Recent ‘Hot Term,’” *Frank Leslie’s Illustrated Newspaper* (Aug. 12, 1882), 390.

61 The Riverside Association at 259 West 69th Street included a bath which required an annual subscription. Of the city’s late-nineteenth-century charitable baths, it was located the closest to one of New York’s Black enclaves, San Juan Hill, which stretched between 60^th^ and 64^th^ Streets and 10^th^ and 11^th^ Avenues. Given the history of racial animosity between Irish and Black residents of the mid-West Side at the turn of the century, it is unclear if and unlikely that racial groups mixed in such spaces. Mayor’s Committee of New York City, *Report on Public Baths and Public Comfort Stations*; New York City Borough of Manhattan Public Works Department, *Statistics Relating to Public Baths and Comfort Stations*, 18; Williams, “New York City’s Public Baths,” 45. On segregation in this era, and the racial violence that enforced it, see Gilbert Osofsky, “Race Riot, 1900: A Study of Ethnic Violence,” *Journal of Negro Education*, 32, No. 1 (Winter, 1963): 16-24.

62 On New York’s non-white populations in 1900, see Ira Rosenwaike, *Population History in New York* City (Syracuse NY, 1972), 66-69, 76–78, 117, 121, 141.

63 Mayor’s Committee of New York City, *Report on Public Baths and Public Comfort Stations*; New York City Borough of Manhattan Public Works Department, *Statistics Relating to Public Baths and Comfort Stations*, 18; Williams, “New York City’s Public Baths,” 45; Burrows and Wallace, *Gotham*, 1198; Wallace, *Greater Gotham*, 550–51. On bather numbers per day, see Buttenwieser, 32.

64 While philanthropic and public bath visits increased across the 1890s, this increase was uneven and at times frustrated reformers. On increasing bathing in the 1890s, see Cross, “Supplement No. 6.”

65 Robert E. Todd, “The Municipal Baths of Manhattan,” *Charities* 19 (1907): 897–99; Williams, “New York City’s Public Baths,” 72–73.

66 Tucker, “Public Baths,” 52.

67 Robert E. Todd, “Four New City Baths and Gymnasiums,” *The Survey* 23 (1910): 682.

68 On New Yorkers’ desire to swim “just for fun” in swimming baths instead of baths equipped with showers and tubs, see Mayor’s Committee of New York City, *Report on Public Baths and Public Comfort Stations*; Committee of Seventy Sub-Committee on Baths and Lavatories, *Preliminary Report of the Sub-Committee on Baths and Lavatories; Prepared in Accordance with a Resolution of the Executive Committee of the Committee of Seventy [*…*]* (New York, 1895), 15.

69 Marta Gutman, “Race, Place, and Play: Robert Moses and the WPA Swimming Pools in New York City,” *Journal of the Society of Architectural Historians* 67, no 4 (2008): 532–561.

70 New York City (Borough of Manhattan), *Public Baths Under the Supervision of the President of the Borough of Manhattan*, 16; Marta Gutman, “Equipping the Public Realm,” in *Robert Moses and the Modern City: The Transformation of New York*, ed. Hilary. Ballon and Kenneth T. Jackson (New York, 2007), 72–85; New York City Department of Parks and Recreation, *History of Parks’ Swimming Pools*, accessed July 3, 2025, https://www.nycgovparks.org/about/history/pools.

71 New York City (Borough of Manhattan), *Public Baths Under the Supervision of the President of the Borough of Manhattan*, 15.

72 “14 Deaths From Heat,” *New York Times*, August 8, 1906.

73 “The Thames Swimming Bath at Charing Cross,” *Illustrated London News*, July 17, 1875.

74 Ian Gordon and Simon Inglis, *Great Lengths: The Historic Indoor Swimming Pools of Britain* (Swindon, 2009).

75 “Lord Rosebery’s Splendid Gift.”

76 Corporation of London, “Ordnance Sheet Showing Swimming Baths around the City” (circa 1895), COL/TSD/PL/02/01/0815, The London Metropolitan Archives.

77 Edgar Harper, *Public Baths and Washhouses, 1907-8* (London, 1909).

78 R.E. Dudgeon, *The Swimming Baths of London* (London, 1870), 27.

79 Harper. *Public Baths and Washhouses, 1907-8*.

80 “London’s Seaside,” *Illustrated London News*, July 29, 1911.

81 “The East-Enders’ Bathing Place: A Scene in Victoria Park,” *Pall Mall Gazette*, September 2, 1893.

82 “The Heat Wave,” *Daily Telegraph*, July 18, 1912.

83 “£25,000 Lido Planned for East End,” *Evening Standard*, December 15, 1934.

84 Laurence Gomme, *Public Baths and Washhouses, 1910-11* (London, 1912).

85 “Bath Queues,” *Daily Mail*, May 15, 1919.

86 Hsuan Hsu, *Air Conditioning* (London, 2024); Eric Dean Wilson, *After Cooling: On Freon, Global Warming, and the Terrible Cost of Comfort* (New York, 2021); “Vacation Days,” *The Crisis* 4, no. 4 (August 1912), 186-188. See also Wiltse, *Contested Waters* and Heather McGhee, *The Sum of Us: What Racism Costs Everyone and How We Can Prosper Together* (New York, 2022).

87 Bronx County Supreme Court, *In the Matter of Acquiring Title by the City of New York to Certain Lands, Lands Under Water and Premises at the Southerly End of Harts Island in the Borough of the Bronx, City of New York, as a Site to be Used for Municipal Purposes, Under the Jurisdiction of the Commissioner of Correction of the City of New York, According to Law, Before: Mr Justice Hatting*, Specialty Term for Trials Supreme Court of the State of New York Bronx County (Feb. 14, 1927), 22. on recreation and segregation in greater New York, see Amanda Martin-Hardin, “The Great Outdoors in Black New York: A History of Erasure, Access, and Activism,” (February 20, 2021), accessed July 3, 2025 https://storymaps.arcgis.com/stories/531a9c2429c94fa6824f29ecbdd354ff.

88 “Jail Escapes Feared as Harlem ‘Coney’ on Hart Island Rises” *New York Evening Post* (June 1, 1925), On Solomon Riley’s attempts to open Black bathing beaches in the east Bronx, see Schlichting, *New York Recentered*, 108–14.

89 Metropolitan Borough of Holborn, “Programme of Opening of Holborn Public Baths and Wash-Houses,” (1902), B/HO/00006/1, Camden Local Studies and Archives Centre.

90 “Le bain des Parisiens. D’une piscine à l’autre.”

91 Nozières, “La piscine élégante,” *Gil Blas*, August 10, 1911.

92 Lemarchand, “Rapport—Au nom de la 6^e^ commission,” 24-30. Esther Da Costa Meyer, *Dividing Paris: Urban Renewal and Social Inequality, 1852–1870* (Princeton, 2022), 149.

93 Gomme, *Public Baths and Washhouses, 1910-11*.

94 Harper, *Public Baths and Washhouses, 1907-8*, 16.

95 On the class and gender divisions of access to water in Paris, see chapter 4 in Meyer, *Dividing Paris*.

96 Préfecture de la Seine, *Annuaire statistique 1900*, 590–91; *Annuaire statistique 1910*, 578–79; *Annuaire statistique 1911*, 578–79; *Annuaire statistique 1921 et 1922*, 416–17.

97 Préfecture de la Seine, *Annuaire statistique 1911*.

98 Naomi Adiv, “The Amphibious Public: A Historical Geography of Municipal Swimming and Bathing New York City, 1870-2013” (PhD diss, City University of New York, 2014).

99 “Chronique. Croquis Parisiens,” *Le Petit Moniteur Universel*, July 19, 1896.

100 Pierre-Charles de Villedeuil, “Questions du jour. Les bains froid,” *La Libert*é, August 15, 1897.

101 “Paris vivant. L’heure du bain,” *Le Soleil*, July 22, 1886. For dead cats and food waste marring the bathing waters at Coney Island, see Ted Steinberg, *Gotham Unbound: The Ecological History of Greater New York* (New York, 2014), 122-3.

102 “Metropolitan Swimming Places,” *Bell’s Life in London*, August 8, 1874.

103 “‘Tenement Memories’ by Comdr. Bernard J. Skahill” (2004), Box 3–9,14 RG 3.7.6, Tenement Museum, New York; “Find Public Baths Endanger Health,” *New York Times*, May 22, 1912; “River Bathhouses Soon to Be Ended,” *New York Times*, June 29, 1912; “New York City Is in Grave Peril from Its Sewage,” *New York Times*, January 11, 1914; On harbor pollution more generally, see Kara Murphy Schlichting, “The Health of the Harbor,” in *Coastal Metropolis: Environmental Histories of Modern New York City*, ed. Carl A. Zimring and Steven H. Corey (Pittsburgh, 2020), 55–67.

104 On eye disease outbreaks, see Tucker, “Public Baths,” 55; Metropolitan Sewerage Commission, *Present Sanitary Condition of New York Harbor and the Degree of Cleanness Which Is Necessary and Sufficient for the Water* (New York, 1912), 29–30; On filtered water use in municipal floating baths, see Gutman, “Equipping the Public Realm,” 74; Adiv, “The Amphibious Public,” 67.

105 “Défense de se baigner,” *L’Avenir*, June 21, 1920; Rachel Mazuy, “Se baigner dans la Seine: plaisir parisien et péril potentiel,” *RetroNews*, September 6, 2023.

106 Préfecture de Police, “Ordonnance concernant les baignades en rivière dans le ressort de la Préfecture de Police, 3 Juillet 1871” (1871), DB/227, Archives de la Préfecture de Police de Paris.

107 “Défense de se baigner.”

108 “Nos lecteurs ont la parole. On réclame des baignades,” *France Soir*, August 13, 1946.

109 Auguste Nardy, “Où donc se baigner? Il n’y a pas de piscine et la ‘pleine eau’ est interdite,” *La Lanterne*, June 21, 1920.

110 “La vague de chaleur,” *La R*é*publique Française*, July 13, 1921.

111 A. Coffignon, “Baignades et natation,” *La Vie Illustr*é*e*, August 31, 1899.

112 Peter S. Soppelsa, “The Fragility of Modernity: Infrastructure and Everyday Life in Paris, 1870-1914,” (PhD diss., University of Michigan, 2009), 305-306.

113 *New York City. Boys Swimming at Dock. Rutgers Slip*, c 1908, photograph, LOT 10832-6, Library of Congress, https://www.loc.gov/item/2007682293/; George Rinhart, *Jumping into the “Old Swimming Hole” at the Foot of Pierpoint Street, in Brooklyn New York*, n.d., photograph, 530849754, Getty Images, https://www.gettyimages.co.uk/detail/news-photo/jumping-into-the-old-swimming-hole-at-the-foot-of-pierpont-news-photo/530849754; *Summertime on the East River*, 1921, photograph, 1921, New York Times photo archive, https://commons.wikimedia.org/wiki/File : Summertime_on_the_East_River, _1921.JPG; William T. Elsing, “Life in New York Tenement-Houses as Seen by a City Missionary,” in *The Poor in Great Cities: Their Problems and What Is Doing to Solve Them* (New York, 1895), 81. For a history of urban swimming and the class and gender aspects of nude male bathing in a North American context see Dale Barbour’s *Undressed Toronto: From the Swimming Hole to Sunnyside, How a City Learned to Love the Beach, 1850-1930* (Winnipeg, 2021).

114 Walt Witman, “Specimen Days,” in *Complete Prose Works: Specimen Days and Collect, November Boughs and Good Bye My Fancy* (New York, 1910), 179.

115 Frank Fowler, “Impressions of the Spring Academy,” *Nation* 84, March 28, 1907, 298, and “Those Who Paint What they See,” *New York Herald*, Feb. 23, 1908, Literary and Art Section, 4. See Marianne Doezema, “The ‘Real’ New York,” in *The Paintings of George Bellows*, eds. Michael Quick, Jane Myers, Marianne Doezema, and Franklin Kelly, introduction John Wilmerding (New York, 1992).

116 Lyman Abbot, “Introduction” in Helen Campell, Thomas W. Knox and Thomas Byrnes, *Darkness and Daylight: or, Lights and shadows of New York life, a pictorial record of personal experiences by day and night in the great metropolis* (Hartford, 1897), 101.

117 Caitlin Davies, *Downstream: A History and Celebration of Swimming the River Thames* (London, 2015).

118 F. Hayman, *Children Swimming in the River Fleet*, 1751, engraving, 10 x 17cm, 1751, SC/PZ/CT/01/3050, The London Metropolitan Archives, https://www.londonpicturearchive.org.uk/view-item?i=318932.

119 Charles Dickens, *Dickens’s Dictionary of the Thames, from Its Source to the Nore: An Unconventional Handbook* (London, 1885), 23.

120 “Summer Season on the Thames,” *Daily Telegraph*, August 19, 1899; “Rowdies on the Thames,” *Daily Telegraph*, August 17, 1904.

121 “Police,” *The Times*, August 4, 1886; “Regent’s Canal Dangers,” *The Times*, May 25, 1914; “Bathers Fined 3d Each,” *Manchester Guardian*, August 29, 1930.

122 *Boys Bathing in a Sluice on the Grand Union Canal*, 1905, photo, 1905, 169058, The London Metropolitan Archives, accessed July 3, 2025, https://www.londonpicturearchive.org.uk/view-item?i=171275.

123 “Regent’s Canal Dangers.”

124 “Heat Wave Kills 5” *New York Times*, June 21, 1923; Hylan made this order again, two years later, in a heat wave: “Connolly Praises Hylan’s ‘Big Heart’,” *New York Times*, June 21, 1925. In Paris and London, fire hydrants were generally located in locked underground cabinets.

125 “Lista de Personas Postradas Por La Asfixia/10 People Perished Yesterday in New York under the Rays of a Melting Sun,” *La Prensa*, June 22, 1923.

126 “Plague’s Wild Work Unchecked,” *The New York Press* (Aug 12. 1896); 1. W.A. Rogers, “Converting a Hydrant into a Shower-Bath While Flushing the Streets with Water in the Crowded Tenement-House Districts,” *Harper’s Weekly*, August 22, 1896; “Heat Wave Kills 5”.

127 “Connolly Praises Hylan’s ‘Big Heart’”; Bain News Service, *N.Y.C. Children in Hot Weather, Playing under Spray of Fire Hydrant, Policemen Standing By*, c 1910, photographic print, Library of Congress, https://www.loc.gov/item/2016648955/; *Enjoying the Croton Surf Under the Fire Department Sprinkler on 85th Street Near Lexington Avenue*, August 17, 1920, photograph, MSS0042_1_160, New York City Municipal Archives; *Queensboro Bridge Playground, Queensboro Bridge Approach*, n.d., photograph, MSS0042_1_160, New York City Municipal Archives.

128 “Nation’s Death List 300,” 2.

129 “Crowds Flee Heat and Sleep in Parks,” *New York Times*, June 7, 1925.

130 “Urchins at Hydrants Give Police a Bath,” *New York Times*, June 10, 1933.

131 *Heat Wave—Trafalgar Square*, c 1910, film ID 2338.28, British Pathé, accessed July 3, 2025, https://www.britishpathe.com/asset/99073/.

132 “80 in the Shade,” *Daily Mail*, June 24, 1905.

133 “Heat Wave Arrives,” *Daily Mail*, July 12, 1901.

134 “Trafalgar Square Fountains,” *Morning Post*, August 8, 1893; “The Week-End Scorch,” *Daily Mirror*, August 11, 1919; “Bathing Boys,” *Daily Mirror*, August 15, 1919.

135 “Heat Wave to Last over Week-End,” *Daily Mirror*, August 10, 1923.

136 “Nation’s Death List 300”; Glen Allen, “How New Yorkers Keep Cool,” *New York Times*, July 23, 1899; any park fountain made a potential pool for city children and young people, see, for example, Bain News Service, *Fountains, Madison Sq. Park on Hot Day*, n.d., glass negative, 5x7in, Library of Congress, https://www.loc.gov/item/2014689800/.

137 “Boys Splash Free in City Hall Fount,” *New York Times*, June 7, 1925.

138 “Heat Kills 21 Here,” *New York Times*, June 6, 1925.

139 For New York City drowning statistics between 1893-1895, see Mayor’s Committee of New York City, *Report on Public Baths and Public Comfort Stations*, 55–57; for drownings during heat waves see, for example, “Heat Clings to City, Killing Five More,” *New York Times*, July 16, 1927; “Sweltering Heat Causes 6 Deaths,” *New York Times*, July 31, 1915.

140 “Drowning Fatality in the Metropolis,” *The Times*, August 26, 1876; “The Drowning Season,” *Daily Mail*, July 31, 1899; “The Drowning Season,” *Daily Mail*, August 2, 1899; “The Drowning Season,” *Daily Mail*, August 14, 1899.

141 “A 6-Year-Old Hero,” *Daily Telegraph*, August 25, 1913.

142 “Regent’s Canal Dangers.”

143 Éric Kocher-Marbœuf, “Les avatars de la noyade au XX^e^ siècle,” in *Corps submergés, corps engloutis: une histoire des noyés et de la noyade de l’Antiquité à nos jours*, ed. Frédéric Chavaud (Saint-Étienne, 2007), 98.

144 Frédèric Chavaud, “Submersions et catastrophes: les figures du noyé au XIX^e^ siécle,” in *Corps sub-mergés*, 75–76.

145 Préfecture de Police, “Un jeune homme noyé dans une baignade à Joinville le Pont” (July 6, 1908); “Noyade en Seine au petit bras de Gennevilliers” (August 10, 1909); “Un jeune homme se noie en Marne, à Joinville le Pont à a baignade du Banc de sable” (August 14, 1910), DA/334, Archives de la Préfecture de Police de Paris.

146 Adiv, “The Amphibious Public,” 57, 125.

147 Richard Keller, *Fatal Isolation: The Devastating Paris Heat Wave of 2003* (Chicago, 2015); Eric Klinenberg, *Heat Wave: A Social Autopsy of Disaster in Chicago*, 2nd ed. (Chicago, 2015).

148 “Urbanus Redux,” *Harper’s*, September 3, 1892.

149 “Dès 2025, la Seine va s’ouvrir à la baignade,” 2024, accessed July 3 2025, https://www.paris.fr/pages/se-baigner-dans-la-seine-en-2024-et-au-dela-6935.

150 “Cool It! NYC,” n.d., accessed July 3, 2025, https://www.nycgovparks.org/about/health-and-safety-guide/cool-it-nyc.

151 “Thames Baths,” 2013, accessed July 3. 2025, http://www.thamesbaths.com/.

